# Formation of Tetrahydrofurano-, Aryltetralin, and Butyrolactone Norlignans through the Epoxidation of 9-Norlignans

**DOI:** 10.3390/molecules25051160

**Published:** 2020-03-05

**Authors:** Patrik A. Runeberg, Dominique Agustin, Patrik C. Eklund

**Affiliations:** 1 Laboratory of Molecular Science and Engineering, Åbo Akademi University, Biskopsgatan 8, 20500 Åbo, Finland; patrik.runeberg@abo.fi; 2 LCC-CNRS, Université de Toulouse, CNRS, UPS, 31077 Toulouse, France; dominique.agustin@iut-tlse3.fr; 3 Institut Universitaire de Technologie Paul Sabatier, Département de Chimie, Av. G. Pompidou, CS20258, F-81104 Castres, France

**Keywords:** lignans, norlignans, epoxidation, molybdenum, *tert*-butyl hydroperoxide, *meta*-chloroperoxybenzoic acid, catalysis

## Abstract

Epoxidation of the C=C double bond in unsaturated norlignans derived from hydroxymatairesinol was studied. The intermediate epoxides were formed in up to quantitative conversions and were readily further transformed into tetrahydrofuran, aryltetralin, and butyrolactone products—in diastereomeric mixtures—through ring-closing reactions and intramolecular couplings. For epoxidation, the classical Prilezhaev reaction, using stoichiometric amounts of *meta*-chloroperbenzoic acid (*m*CPBA), was used. As an alternative method, a catalytic system using dimeric molybdenum-complexes [MoO_2_L]_2_ with ONO- or ONS-tridentate Schiff base ligands and aqueous *tert*-butyl hydroperoxide (TBHP) as oxidant was used on the same substrates. Although the epoxidation was quantitative when using the Mo-catalysts, the higher temperatures led to more side-products and lower yields. Kinetic studies were also performed on the Mo-catalyzed reactions.

## 1. Introduction

**Figure 1:**
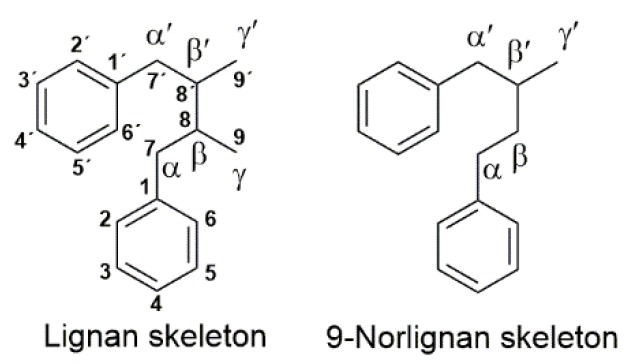
The structure of lignan and 9-norlignan and norlignan.

In 1936, Haworth introduced lignans as a group of plant-based compounds consisting of β-β′-linked dimeric phenylpropane units (Figure 1) [1]. Similar to other natural phenolics such as stilbenes and flavonoids, lignans are formed through the shikimic acid pathway [2]. Lignans have attracted great interest in research as they have a number of health benefits, such as being strong antioxidants, lowering the risk of coronary heart disease, and having neuroprotective effects and anticancer properties [3,4,5,6]. Lignans are also found in foods, for example, flaxseed, sesame, chickpeas and cereals (rye, oat, barley), and red wine contains high levels of lignans. [7] 

A subclass of lignans called norlignans are dimers composed of a phenylpropane unit and a phenylethane unit coupled through a β-β′ linkage (Figure 1). The prefix “nor” indicates the lack of one or more carbon in the parent skeleton, and the term 9-norlignan is used when the lignan structure lacks C-9 (or γ carbon). As lignans, norlignans are also found in plants, having a range of biological activity [8,9]. 

**Figure 2:**
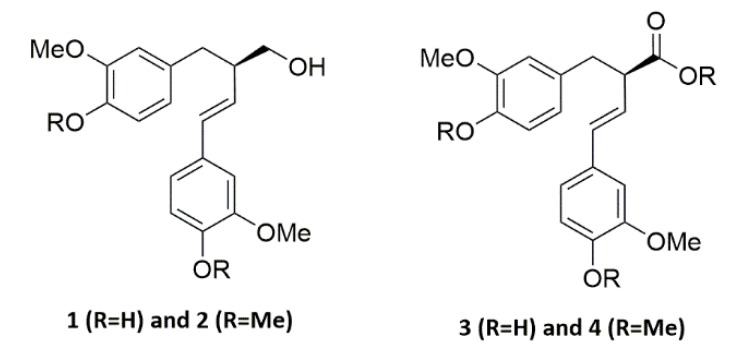
Imperanene (**1**), dimethyl-imperanene (**2**), Imperaneic acid (**3**), permethylated imperaneic acid (**4**).

The lignan 7-hydroxymatairesinol (HMR) can be isolated in large-scale from the knotwood of Norway spruce (*Picea abies*) as it constitutes up to 24% of the dry weight [10]. HMR can be used as raw material for the semi-synthesis of a range of different lignans, among others are a family of norlignans belonging to the imperanene family [9]. Some of these semi-synthetic and unsaturated 9-norlignans were used as substrates in this study (Figure 2, substrates **1-4**). The substrates were chosen for two reasons. Firstly, they were chosen to study the selectivity in the oxidation reactions (epoxidation) with substrates containing free phenolics (**1** and **3**) compared to protected (methoxylated) derivatives (**2** and **4**). Secondly, we wanted to study the outcome of the epoxidation reaction in the presence of a primary hydroxyl (**1**-**2**), carboxylic acid (**3**) and methyl ester (**4**).

Epoxidation reactions are undoubtedly important in organic chemistry and several methods have been developed to perform these reactions [11,12,13,14]. However, to our knowledge, no epoxidation reactions have previously been reported for substrates **1**–**4**. As part of our work on oxidative transformation of lignans and norlignans, we wanted to investigate how these substrates react upon epoxidation. We chose two different methods for this study. First, the stoichiometric *m*CPBA-mediated epoxidation method known as the Prilezhaev reaction was studied. Previously, *m*CPBA has been used for Baeyer-Villiger oxidation of lignans containing ketone or aldehyde groups, for epoxidation of lignan structures containing double bonds, and for epoxidation of dihydro-naphthalene model compounds having a norlignan-type skeleton [15,16]. Because of the simplicity of the Prilezhaev reaction and the average high product yields for the reaction, *m*CPBA was a good epoxidation agent for the purpose of our study.

As we wanted to study more environmentally friendly alternatives for epoxidation, our attention turned to catalytic reactions using H_2_O_2_, *tert*-butyl hydroperoxide (TBHP), or even O_2_ as oxidants. Since the 1960s, numerous complexes of molybdenum have been used as catalysts for efficient epoxidation of olefins together with hydroperoxides [17]. More recently, scientists have further developed molybdenum complexes for this purpose [18]. Some complexes have attracted our interest, i.e., [MoO_2_L] complexes (L being a tridentate Schiff base ligand) since their synthesis is quite easy [19,20]. These complexes have a long shelf life and have been shown to exhibit interesting reactivity under green conditions. For example, epoxidations of cyclooctene and cyclohexene [19,20,21,22] and the natural substrate limonene has been demonstrated [23]. It has also been shown that epoxidation of sesquiterpenes could be achieved using TBHP as oxidant, and the reactivity was compared to *m*CPBA [24]. As the substrates previously studied for [MoO_2_L-TBHP] epoxidations were fairly simple and without functional groups, it was interesting to investigate how this Mo-catalyzed epoxidation could work on the more complex substrates **1**–**4**.

**Scheme 1:**
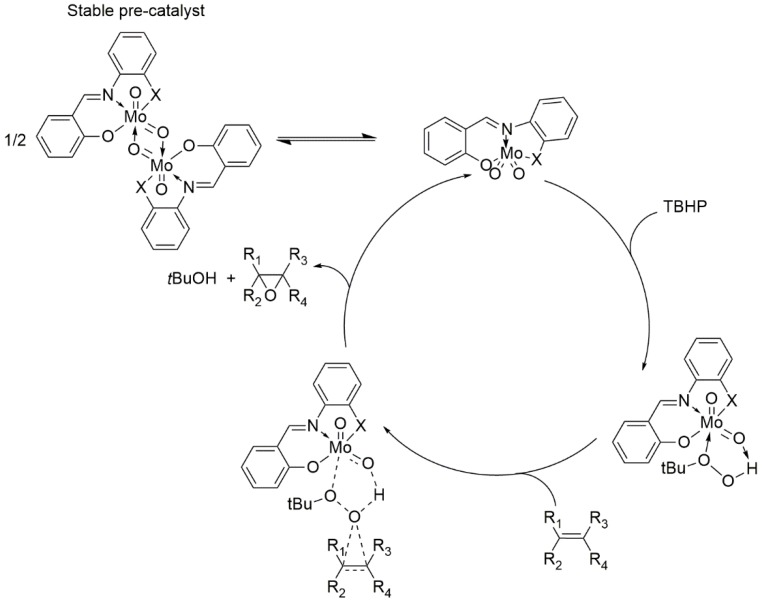
The dimeric Mo(VI)-complex (pre-catalyst), with salicylideneaminophenolato ([MoO_2_(SAP)]_2_) (X = O) or salicylideneaminothiophenolato ([MoO_2_(SATP)]_2_) (X = S) tridentate ligands and the formation of the monomeric active catalysts. The active [MoO_2_L] species is coordinated to TBHP followed by Bartlett-type epoxidation of olefins [19].

The mechanism for the [MoO_2_L-TBHP] epoxidations can be explained through a Bartlett-like mechanism, implying the formation of a pentacoordinated species [MoO_2_L] followed by the coordination of TBHP (Scheme 1). The [MoO_2_L-TBHP] adduct was responsible of the oxygen transfer from the oxidant to the olefin. [19]

The aim of this study was to evaluate the traditional *m*CPBA-reaction and compare it to the novel Mo-catalytic version for epoxidation of the presented substrates. Furthermore, the subsequent reactions of the epoxy-intermediates to form different norlignan structures were the target of investigation.

## 2. Results and Discussion

**Scheme 2:**
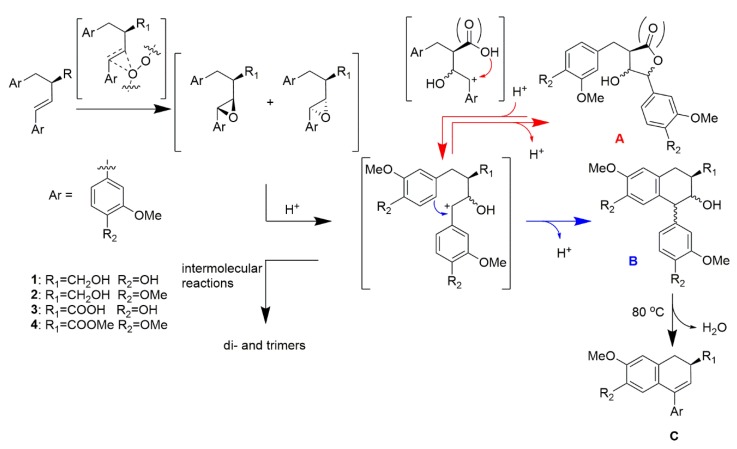
Peroxide mediated epoxidation of the double bond in norlignans. The formed epoxide intermediates were most likely opened to the benzylic cation intermediate, which through nucleophilic attack (from hydroxyl, carboxyl or aryl group) formed products A and B. At 80 °C partial water elimination of product B gave the unsaturated product C. The cationic intermediate also reacted through intermolecular couplings to oligomers.

All norlignans reacted at the double bond forming the epoxide-intermediate as two diastereomers. The formed epoxides were subject to rapid intramolecular nucleophilic attack to give the final products (Scheme 2, products A and B). Since both stereoisomers of the epoxides were formed, and the subsequent nucleophilic attacks were not stereospecific, four possible stereoisomers were formed. However, some diastereomers were formed only in trace amounts. 

In addition, during the transformations product A was probably partially re-opened to the cationic intermediate and eventually transformed into the thermodynamic product B by the nonreversible Friedel-Craft type ring closure. In fact, we expected product A to be quantitatively converted into product B under acidic conditions, as similar pH dependent C-O bond openings and product transformations have been reported for other lignans [25]. During the reactions we could not clearly detect the transformation of A to B but upon treatment with excess acid the conversion was quantitative.

In Table 1, the yields for products and side products are shown for each reaction.

### 2.1. m-CPBA-Mediated Epoxidation

The Prilezhaev reaction at room temperature gave quantitative conversions within 30 min for all substrates. No epoxides were detected on GC-MS nor NMR, as they rapidly reacted to product A and B (and a mixture of minor products). 

The reaction worked well with free phenolic groups as **1** had a quantitative conversion to products **1A** (isolated yield of 60% of four isomers) and **1B** (isolated yield of 12% of two isomers) within 30 min at room temperature. Substrate **2** with protected phenolics (as methoxyl groups) also gave a quantitative conversion within 20 min at room temperature to products **2A** (42% isolated yield as four isomers) and **2B** (46% isolated yield as four isomers).

The Prilezhaev reaction gave 96% conversion of **3** after 30 min at room temperature to a reaction mixture with a product distribution, according to NMR, of 71% **3A** (total isolated yield for the four isomers was 51%) and 24% **3B** (total isolated yield for two isomers was 19%). Furthermore, the isolated product **3A** could quantitatively be converted to **3B** by stirring in trifluoroacetic acid (TFA) at room temperature for 20 min. Thus, a final isolated yield of 70% of **3B** could be reached.

**Figure 3:**
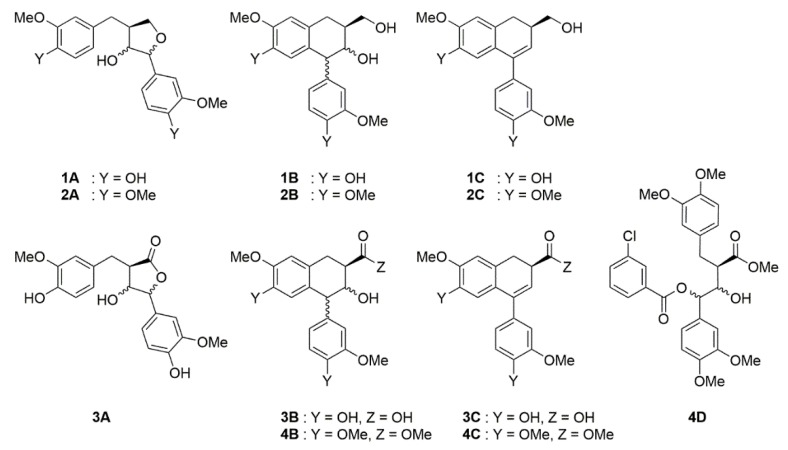
Products formed in the reactions.

The Prilezhaev reaction of **4** using one equivalent *m*CPBA for 30 min resulted in an 83% conversion. Two products were observed, product **4B** and **4D**, (a 7-*m*CBA-coupled adduct, Figure 3) in a 4:6 ratio. Substrate **4** could not react by pathway A, and the nucleophilic attack by *m*CBA was competing with the slower pathway **B**. This type of reaction has been reported previously [26]. The byproduct **4D** could, however, be quantitatively hydrolyzed to **4B** by stirring in TFA at room temperature for 20 min.

### 2.2. Mo-Catalyzed Epoxidation with TBHP

Most substrates were quantitatively converted by the Mo-catalysts. However, the higher temperatures resulted in partial water elimination of product B to a new unsaturated structure as seen for product C in Scheme 2. This side product was identified and quantified using GC-MS and NMR but was not further isolated and characterized as only small amounts were formed (≤7%). Additionally, some unidentified oligomeric side products were observed. The oligomers were likely formed through intermolecular reactions of the epoxide intermediates, as the double bond seemed to be reacting to products containing alcohol and ether functions. Analyses using high pressure size exclusion chromatography (HPSEC) revealed that the oligomers consisted mostly of di- and trimers. At 80 °C, traces of benzaldehydes were also formed through oxidative cleavage of double bond. Due to these various side reactions, the yield of products A and B were moderate, although the conversions to epoxides were quantitative (Table 1). 

For the Mo-catalyzed reactions, no or very slow reactions were observed with less than 5 equivalents of TBHP or with reaction temperatures lower than 80 °C, which restricted the optimization of this reaction by lowering the reaction temperature. As a consequence, lower yields of the final products were obtained using MoO_2_L complexes performed at 80 °C (20%–40% formation of products **A** and **B** according to NMR of the reaction mixtures) compared to reactions with *m*CPBA operated at room temperature (>90% formation of products **A** and **B**).

The resulting reaction mixtures for each substrate were similar in the processes using both catalysts [MoO_2_(SAP)]_2_ and [MoO_2_(SATP)]_2_. However, the reaction rates were higher with [MoO_2_(SATP)]_2_. This observation was in agreement with previous kinetic studies with both catalysts for epoxidation of cyclooctene, under solvent-free conditions with TBHP at 80 °C [20]. Substrate 1 reacted to identical reaction mixtures with both Mo-catalysts (Entries 1 and 2). As a side note, analyses of these reactions were done at one and three hours, and quantitative conversion for the [MoO_2_(SATP)]_2_-catalyzed reaction (Entry 3) was reached in a shorter time compared to the [MoO_2_(SAP)]_2_-catalyzed reaction (Entry 2).

In the absence of Mo-catalyst, even a large excess (10 equivalents) of TBHP did not react with substrate **1**, exhibiting the role of the MoO_2_L complexes as catalyst.

**Figure 4:**
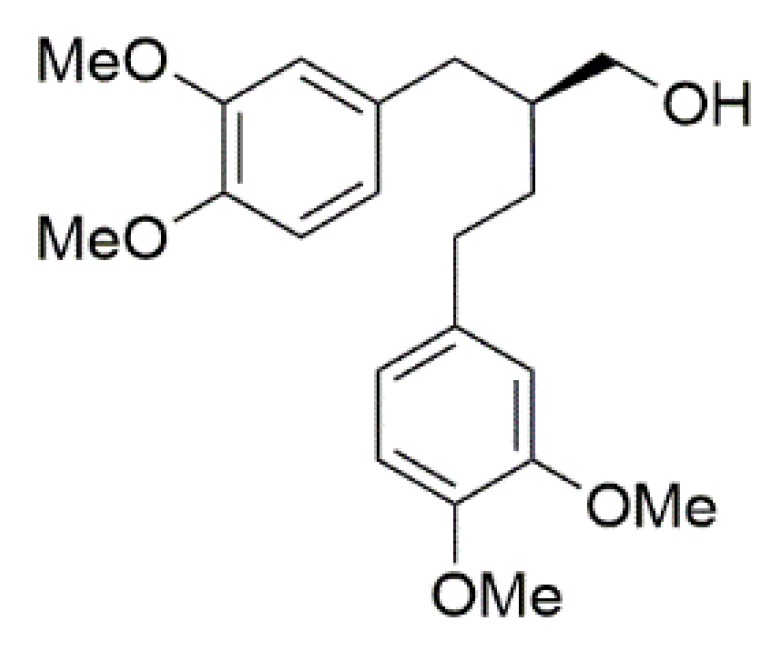
Dimethyl-dihydroimperanene, used as internal standard in the kinetic studies.

**Figure 5:**
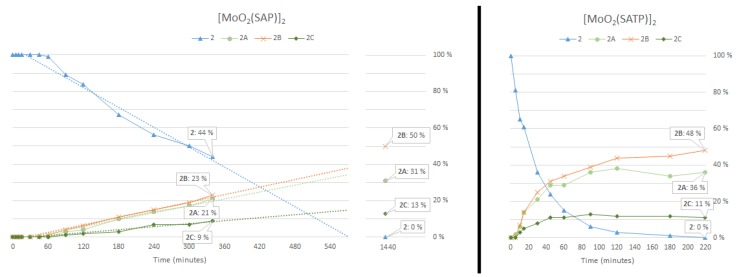
Kinetic studies on the reaction rate of **2** in toluene with 0.5 mol% dimeric precatalyst ([MoO_2_(SAP)]_2_ to the left and [MoO_2_(SATP)]_2_ to the right, and 5 equivalents of TBHP (70 wt% in water) at 80 °C. The trend lines (stacked lines) for [MoO_2_(SAP)]_2_ suggested that the reaction had a quantitative conversion after 570 min.

To further investigate this, reaction kinetic studies were performed with substrate **2** (14.5 mM) in toluene, using 0.5 mol% dimeric Mo(VI)-precatalyst (i.e. 1% mol Mo) and 5 equivalents of TBHP (70 wt% in water) at 80 °C. The reaction was followed by GC-MS using dimethyl-dihydroimperanene as internal standard (Figure 4). Samples were taken at specific time intervals and dried in a vacuum. Derivatization of the samples was done using tetramethyldisilazane and chlorotrimethylsilane in pyridine prior to GC-MS analysis [27]. The results showed that the reaction catalyzed by [MoO_2_(SATP)]_2_ was faster (as mentioned previously), with a quantitative conversion reached after 3 h 40 min (Figure 5). For comparison, at 3 h 40 min, the [MoO_2_(SAP)]_2_-catalyzed reaction showed a conversion of less than 50% and, calculated from the trend of the reaction rate, a quantitative conversion was reached after approximately 9 h 30 min. The rate was 2.6 times slower than the [MoO_2_(SATP)]_2_-catalyzed reaction. For both catalysts, the final reaction mixture gave products **2A**, **2B** and **2C** in roughly 3:5:1 ratio. Quantification using GC analyses showed a total conversion to products **2A**, **2B** and **2C** at around 95%, for both reactions. Quantification using NMR analysis, however, indicated that the total yield (**2A**, **2B** and **2C**) was around 70% (calculated from the ratio of integrals in the ^1^H spectrum). This difference may be explained by the fact that signal intensity in the total ion chromatogram is not fully quantitative, due to a different degree of ionization for the products. As each product was not quantitatively calibrated on GC, quantification using NMR seemed to be more reliable.

Furthermore, the NMR spectra showed that the ratio of products **2A**, **2B** and **2C** was roughly 6:2:1. The variation in product distribution between GC-MS and NMR may be partially explained by transformation of **2A** to **2B** during the derivatization step or upon injection to the GC-MS. Those types of transformations have been reported for other lignan structures as well [25].

For substrate **3** (containing OH functions), the Mo-catalyzed reactions gave a mixture of mono- and oligomeric products. Only traces of the expected ring closed products (**3A** and **3B**) were detected using NMR and GC-MS. NMR analyses of the reaction mixtures showed that the double bond had reacted. Here, again, the high temperature in these reactions may result in unselective reactions of the epoxide intermediate. With lower equivalents of TBHP, no conversion occurred even with higher equivalents of pre-catalysts (Entries 8 and 10).

**Figure 6:**
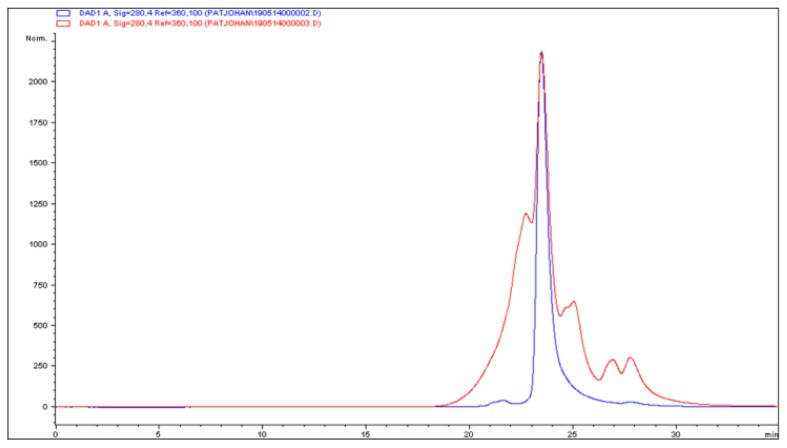
Size exclusion chromatogram of substrate **4** (blue). The reaction mixture after 21 hours with 5 equivalents TBHP and 1.5 mol% ([MoO_2_(SAP)]_2_) in toluene at 80 °C (red). Mono-, dimers and small amounts of trimers are shown between 20 and 24 min. The smaller fragments correspond, in addition to 3,4-dimethoxybenzaldehyde, to molybdenum complexes (24–30 min).

Substrate **4** reacted slower compared to the other norlignans. NMR showed the conversion to one major product (**4B**) in 14% or 19% for [MoO_2_(SAP)]_2_ or [MoO_2_(SATP)]_2_ respectively, as well as other mono- (**4C**, **4D** and other unknown products) and oligomers. Analysis using HPSEC gave a rough estimation of 40% monomeric structures, 35% di- and trimers, and 25% small molecular structures including molybdenum complexes (Figure 6). GC-MS revealed that the main degradation product was 3,4-dimethoxybenzaldehyde issued from the C=C oxidative cleavage.

## 3. Conclusions

The results suggest that all studied norlignans reacted in up to quantitative conversion to epoxide intermediates using *m*CPBA or by Mo-catalyzed reactions with TBHP as oxidant. However, the intermediate products rapidly reacted further through nucleophilic attack at the epoxides forming tetrahydrofuran or lactone structures (A) or aryltetralin (B) structures. The main products were formed by route A (Product A in Scheme 2), but the arylic ring closure at C-6′-position (Product B in Scheme 2) was a competing reaction. Product A could also be further transformed to B by treatment in acidic conditions. As a result of high temperatures, longer reaction times, and higher equivalents of peroxide, the Mo-catalyzed reactions also gave other mono- and oligomeric (di- and trimeric) side products resulting in lower yields of defined products (A and B). The results, however, showed that these Mo-catalysts can successfully be used for epoxidation of more complex substrates than previously reported, as long as the products are stable at those reaction conditions. Once optimized, the advantage of the MoO_2_L/TBHP procedures could lie in the fact that less post-reaction procedures are needed, interesting in terms of mass efficiency, and that TBHP is lighter than *m*-CPBA, interesting in terms of atom economy.

## 4. Materials and Methods 

### 4.1. Materials

All solvents were commercially available and used as supplied by the supplier (Merck KGaA, Darmstadt, Germany). NMR solvents were purchased from VWR Chemicals (CD_3_OD, CDCl_3_). All norlignan substrates were synthesized from the natural lignan hydroxymatairesinol at Johan Gadolin Process Chemistry Center, Åbo Akademi University [9]. The [MoO_2_(SAP)]_2_ and [MoO_2_(SATP)]_2_ complexes were synthesized according to published procedures at the IUT-LCC, Castres. [20]

### 4.2. Methods

#### 4.2.1. Prilezhaev Reaction (mCPBA)

In a typical reaction, the substrate (1.0 equivalent) was dissolved in DCM (50 mM lignan concentration). Under stirring, *m*CPBA (1.0 equivalent) was added and the reaction continued at room temperature for 20–30 min. Aqueous saturated NaHCO_3_ was added and the organic phase was separated. The aqueous phase was extracted with EtOAc. The combined organic phases were dried using Na_2_SO_4_, filtered, and concentrated in a vacuum. The isolated products were retrieved through purification using column chromatography with silica gel 60 as stationary phase and chloroform and methanol as eluents.

#### 4.2.2. Mo-Catalyzed Epoxidation by TBHP

In a typical reaction, the substrate (1.0 equivalent) and molybdenum-catalyst (0.5 mol% of dimer) was dissolved in toluene (50 mM substrate concentration). The solution was heated to 80 °C, followed by dropwise addition of aqueous TBHP (70 wt%, 5 equivalents). The reaction mixture was stirred at 80 °C in an open air atmosphere. After an appropriate time (1–20 h) the reaction mixture was quenched by addition of excess solid MnO_2_, cooled to room temperature, and a dark precipitate (Mo-complex + MnO_2_) was filtered off. The crude mixture was concentrated in a vacuum. The isolated products were retrieved through purification using column chromatography with silica gel 60 as stationary phase and chloroform and methanol as eluents.

#### 4.2.3. Analytical Methods

HRMS of the negatively charged deprotonated products (positively charged Na-adducts of **4B**) were recorded on a Bruker Daltonics micrOTOF-Q instrument using an electrospray ionization/quadrupole/time-of-flight systems.

^1^H and ^13^C-NMR spectra were recorded on a Bruker AV 500 spectrometer at 500 and 125 MHz, respectively. 2D-experiments were recorded using standard pulse sequences, and the chemical shifts are reported downfield from tetramethylsilane. 

GC-EIMS analyses were performed on an Agilent Technologies 7890A GC-system equipped with a 5975C EIMS-detector and an Agilent J&W HP-5ms GC Column (30 m × 0.25 mm, 0.25 μm film) (Agilent Technologies Inc., Santa Clara, CA, USA).

HP-SEC analyses were performed on an Agilent 1100 Series HPLC instrument equipped with a G1315B DAD-detector, 2 × Jordi Gel DVB 500A (300 mm × 7.8 mm) columns (Columnex LLC, New York, NY, USA; 40 °C), and a 50 mm × 7.8 mm guard column. One percent of AcOH in THF served as eluent at a flow rate of 0.8 mL/min.

#### 4.2.4. Isolation of Products

The products were isolated from the reaction mixtures using a Teledyne ISCO combiFlash^®^ EZ Prep UV/ELSD-unit (Teledyne ISCO, Lincoln, NE, USA), with RediSep Rf gold silica flash chromatography columns (20–40 μm). A chloroform/methanol eluent system was used, starting from 0 vol% methanol and gradually over 45 min increasing the methanol ratio to 15 vol%.

### 4.3. Experimental Data

The experimental data for the major isomers of all products are listed here. The various diastereomers of each product were not separated through column chromatography, and only the major diastereomers could be characterized using NMR (see Supplementary Materials). Furthermore, the methoxyl-signals in ^1^H-NMR spectra were overlapped with the signals for H-8 in **1A** and **2A** and 7-H and 8-H in **1B**, **2B**, **3B**, and **4B**, and the stereochemistry could therefore not be solved for these products. On the other hand, the stereochemistry for all isomers of **3A** was solved.

#### 4.3.1. Product **1A**

Product **1A** was a white solid with an isolated yield of 60%.

Major isomer of Product **1A**:

^1^H-NMR (500 MHz, CDCl_3_): δ (ppm) = 6.92 (d, *J* = 1.6 Hz, 1 H, H-2), 6.89 (2 H, H-5; H-6), 6.83 (1 H, H-5′), 6.67 (1 H,H-6′), 6.64 (1 H, H-2′), 5.64 (s, 1 OH), 5.55 (s, 1 OH), 4.55 (d, *J* = 6.6 Hz, 1 H, H-7), 4.14 (dd, *J* = 8.8, 7.8 Hz, 1 H, H-9′a), 3.89 (s, 3 H, OMe), 3.86 (dd, *J* = 8.8, 6.5 Hz, 1 H, H-9′b), 3.85 (s, 3 H, OMe), 3.83 (m, 1 H, H-8), 2.79 (dd, *J* = 13.8, 7.2 Hz, 1 H, H-7′a), 2.66 (dd, *J* = 13.8, 8.2 Hz, 1 H, H-7′b), 2.54 (m, 1 H, H-8′).

^13^C-NMR (125 MHz, CDCl_3_): δ (ppm) = 146.6, 146.5, 145.2, 144.1, 132.3, 131.5, 121.2, 118.8, 114.5, 114.3, 111.1, 108.3, 86.2, 83.2, 71.8, 56.0, 55.9, 49.0, 37.5.

HRMS: found 345.1307 (M^−^). C_19_H_21_O_6_^−^ requires 345.1344. 

#### 4.3.2. Product **1B**

Product **1B** was a white solid with an isolated yield of 12 %.

Major isomer of Product **1B**:

^1^H-NMR (500 MHz, CDCl_3_): δ (ppm) = 6.91 (d, *J* = 8.1 Hz, 1 H, H-5), 6.74 (dd, *J* = 8.1, 1.8 Hz, 1 H, H-6), 6.65 (d, *J* = 1.8 Hz, 1 H, H-2), 6.57 (s, 1 H, H-2′), 6.27 (s, 1 H, H-5′), 3.77–3.90 (m, 4 H, H-7; H-8, H-9′a; H-9′b), 3.86 (s, 3 H, OMe), 3.85 (s, 3 H, OMe), 2.82 (dd, *J* = 16.3, 5.3 Hz, 1 H, H-7′a), 2.66 (dd, *J* = 16.3, 12.5 Hz, 1 H, H-7′b), 2.24 (m, 1 H, H-8′).

^13^C-NMR (125 MHz, CDCl_3_): δ (ppm) = 147.2, 145.4, 145.2, 144.0, 133.5, 131.0, 126.5, 122.9, 115.1, 114.8, 111.6, 110.2, 79.3, 67.5, 56.1, 56.1, 55.4, 42.3, 31.5.

HRMS: found 345.1314 (M^−^). C_19_H_21_O_6_^−^ requires 345.1344.

#### 4.3.3. Product **2A**

Isolated yield of three stereoisomers of **2A** was 44%. White solid.

Major isomer of product **2A**:

^1^H-NMR (500 MHz, CDCl_3_): δ (ppm) = 6.91–6.95 (m, 2 H), 6.84 (d, *J* = 8.7 Hz, 1 H), 6.77 (d, *J* = 8.0 Hz, 1 H), 6.69 (dd, *J* = 8.0, 1.9 Hz, 1 H), 6.66 (d, *J* = 1.9 Hz, 1 H), 4.57 (d, *J* = 6.5 Hz, 1 H), 4.14 (dd, *J* = 8.7, 7.7 Hz, 1 H), 3.87 (s, 3 H), 3.86 (1 H), 3.86 (s, 3 H), 3.83–3.85 (m, 7 H), 2.81 (dd, *J* = 13.7, 6.9, 1 H), 2.65 (dd, *J* = 13.7, 8.7, 1 H), 2.55 (m, 1 H).

^13^C-NMR (125 MHz, CDCl_3_): δ (ppm) = 149.1, 149.0, 148.6, 147.6, 133.0, 132.2, 120.5, 118.1, 111.9, 111.4, 111.1, 108.8, 86.2, 83.1, 71.8, 55.9, 55.9, 55.9, 55.8, 49.0, 37.3.

HRMS: found 373.1619 (M^−^). C_21_H_25_O_6_^−^ requires 373.1657.

#### 4.3.4. Product **2B**

Isolated yield of **2B** was 48 %. White solid.

Major isomer of product **2B**:

^1^H-NMR (500 MHz, CDCl_3_): δ (ppm) = 6.86 (d, *J* = 8.2 Hz, 1 H, H-5), 6.81 (dd, *J* = 8.2, 1.9 Hz, 1 H, H-6), 6.66 (d, *J* =1.9 Hz, 1 H, H-2), 6.59 (s, 1 H, H-2′), 6.18 (s, 1 H, H-5′), 3.89 (s, 3 H, OMe), 3.83–3.86 (m, 2 H, H-7; H-8), 3.85 (s, 3 H, Ome), 3.81 (s, 3 H, Ome), 3.88–3.81 (m, 2 H, H-9′a; H-9′b), 3.58 (s, 3 H, Ome), 2.82 (dd, *J* = 16.4, 5.4 Hz, 1 H, H-7′a), 2.67 (dd, *J* = 16.4, 12.5 Hz, 1 H, H-7′b), 2.22 (m, 1 H, 8′).

^13^C-NMR (125 MHz, CDCl_3_): δ (ppm) = 149.3, 148.3, 147.7, 147.3, 134.6, 129.9, 127.2, 122.3, 112.2, 112.0, 111.2, 110.7, 79.0, 67.1, 55.9, 55.9, 55.9, 55.8, 55.2, 42.0, 31.3.

HRMS: found 373.1636 (M^−^). C_21_H_25_O_6_^−^ requires 373.1657.

#### 4.3.5. Products **3A**

Isolated yield of four diastereomers (isomers 1, 2, 3, 4 in a 12:7:6:5 ratio) of **3A** was 51%. White solid.

##### Isomer 1 of **3A** (7-(*R*) 8-(*R*)):

^1^H-NMR (500 MHz, MeOD): δ (ppm) = 6.87 (d, *J* = 1.6 Hz, 1 H, H-2′), 6.81 (d, *J* = 2.0 Hz, 1 H, H-2), 6.78 (d, *J* = 8.2 Hz, 1 H, H-5), 6.69 (dd, *J* = 8.2, 2.0 Hz, 1 H, H-6), 6.68–6.69 (m, 2 H, H-5′ and H-6′), 5.33 (br. s, 1 H, H-7), 4.20 (dd, *J* = 5.3, 1.0 Hz, 1 H, H-8), 3.81 (s, 3 H, OMe), 3.79 (s, 3 H, OMe), 2.93–2.96 (m, 2 H, H-7′ab), 2.84–2.88 (m, 1 H, H-8′).

^13^C-NMR (125 MHz, MeOD): δ (ppm) = 179.7 (9′), 149.3, 148.8, 147.6, 145.9, 132.0, 129.5, 122.3, 118.4, 116.4, 116.1, 113.7, 109.6, 88.8, (7), 75.6 (8), 56.4 (OMe), 56.3 (OMe), 46.6 (8′), 30.0 (7′). 

HRMS: found 359.1143 (M^−^). C_19_H_19_O_7_^−^ requires 359.1136.

##### Isomer 2 of **3A** (7-(*S*) 8-(*R*)):

^1^H-NMR (500 MHz, MeOD): δ (ppm) = 6.97 (d, *J* = 0.7 Hz, 1 H, H-2′), 6.90 (d, *J* = 1.9 Hz, 1 H, H-2), 6.80 (m, 2 H, H-5′ and H-6′), 6.75 (dd, *J* = 8.0, 1.9 Hz, 1 H, H-6), 6.71 (d, *J* = 8.0 Hz, H-5), 5.31 (d, *J* = 2.8 Hz, 1 H, H-7), 4.26 (dd, *J* = 4.5, 2.8 Hz, 1 H, H-8), 3.86 (s, 3 H, OMe), 3.83 (s, 3 H, OMe), 3.14 (ddd, *J* = 11.0, 4.5, 3.9 Hz) 3.00 (dd, *J* = 13.9, 3.9 Hz, 1 H, H-7′a), 2.94 (dd, *J* = 13.9, 11.0 Hz, 1 H, H-7′b).

^13^C-NMR (125 MHz, MeOD): δ (ppm) = 180.0 (9′), 148.9 (3′), 148.8 (3), 147.7 (4′), 146.0 (4), 132.4 (1′), 127.4 (1), 122.3 (6), 121.0 (6′), 116.2 (5), 115.8 (5′), 113.6 (2), 111.9 (2′), 86.0 (7), 72.8 (8), 56.4 (OMe), 56.3 (OMe), 51.1 (8′), 30.2 (7′). 

HRMS: found 359.1143 (M^−^). C_19_H_19_O_7_^−^ requires 359.1136.

##### Isomer 3 of **3A** (7-(*R*) 8-(*S*)):

^1^H-NMR (500 MHz, MeOD): δ (ppm) = 6.83 (d, *J* = 1.9 Hz, 1 H, H-2′), 6.65-7.75 (m, 4 H, H-5, H-6, H-5′, H-6′), 6.51 (d, *J* = 2.0 Hz, 1 H, H-2) 4.93 (d, *J* = 7.2 Hz, 1 H, H-7), 4.06 (dd, *J* = 8.6, 7.2 Hz, 1 H, H-8), 3.80 (s, 3 H, OMe), 3.71 (s, 3 H, OMe), 3.11 (dd, *J* = 13.7, 4.3, 1 H, H-7′a), 2.99 (ddd, *J* = 8.6, 5.7, 4.3 Hz, 1 H, H-8′), 2.93 (dd, *J* = 13.7, 5.7 Hz, 1 H, H-7′b). 

^13^C-NMR (125 MHz, MeOD): δ (ppm) = 178.1 (9′), 149. 2 (3′), 148.9 (3), 148.2 (4′), 146.4 (4), 130.4 (1′), 130.0 (1), 123.4 (6), 121.0 (6′), 116.1 (5), 115.9 (5′), 114.3 (2), 110.3 (2′), 86.8 (7), 77.7 (8), 56.3 (OMe), 56.3 (OMe), 51.4 (8′), 33.1 (7′).

HRMS: found 359.1143 (M^−^). C_19_H_19_O_7_^-^ requires 359.1136.

##### Isomer 4 of **3A** (7-(*S*) 8-(*S*)):

^1^H-NMR (500 MHz, MeOD): δ (ppm) = 6.90 (d, *J* = 0.9 Hz, 1 H, H-2′), 6.89 (d, *J* = 1.9 Hz, 1 H, H-2), 6.79 (d, *J* = 8.1 Hz, 1 H, H-5), 6.76 (m, 2 H, H-5′ and H-6′), 6.74 (dd, *J* = 8.1, 1.9 Hz, 1 H, H-6), 5.32 (d, *J* = 4.4 Hz, 1 H, H-7), 4.28 (dd, *J* = 4.4, 2.4 Hz, 1 H, H-8),3.86 (s, 3 H, OMe), 3.84 (s, 3 H, OMe), 3.02 (dd, *J* = 13.9, 5.7 Hz, 1 H, H-7′a), 2.96 (dd, *J* = 13.9, 8.2 Hz, 1 H, H-7′b), 2.90 (ddd, *J* = 8.2, 5.7, 2.4 Hz, 1 H, H-8′).

^13^C-NMR (125 MHz, MeOD): δ (ppm) = 180.4 (9′), 149.1 (3), 148.8 (3′), 147.8 (4′), 146.6 (4), 130.6 (1′), 127.0 (1), 122.7 (6′), 120.9 (6), 116.3 (5), 115.9 (5′), 113.7 (2′), 111.9 (2), 85.5 (7), 74.4 (8), 56.4 (OMe), 56.4 (OMe), 53.1 (8′), 34.3 (7′). 

HRMS: found 359.1143 (M^−^). C_19_H_19_O_7_^−^ requires 359.1136.

#### 4.3.6. Products **3B**

Isolated yield of **3B** as two diastereomers was 19%. White solid.

##### Isomer 1 of **3B**:

^1^H-NMR (500 MHz, MeOD): δ (ppm) = 6.75 (d, *J* = 8.0 Hz, 1 H, H-5), 6.70 (d, *J* = 2.0 Hz, 1 H, H-2), 6.65 (dd, *J* = 8.0, 2.0 Hz, 1 H, H-6), 6.64 (s, 1 H, H-2′), 6.15 (s, 1 H, H-5′), 4.04 (dd, *J* = 10.5, 9.8 Hz, 1 H, H-8), 3.80 (s, 3 H, OMe), 3.78 (s, 3 H, OMe), 3.71 (d, *J* = 9.8 Hz, 1 H, H-7), 3.13 (dd *J* = 16.2, 12.2 Hz, 1 H, H-7′a), 3.00 (dd, *J* = 16.2, 5.0 Hz, 1 H, H-7′b), 2.80 (ddd, *J* = 12.2, 10.5, 5.0 Hz, 1 H, H-8′).

^13^C-NMR (125 MHz, MeOD): δ (ppm) = 178.1, 148.9, 147.6, 146.2, 145.8, 136.6, 132.4, 126.4, 123.5, 117.1, 116.0, 114.0, 111.7, 76.0, 56.3, 56.3, 55.2, 50.4, 33.4.

HRMS: found 359.1144 (M^−^). C_19_H_19_O_7_^−^ requires 359.1136.

##### Isomer 2 of **3B**

^1^H-NMR (500 MHz, MeOD): δ (ppm) = 6.75 (s, 1 H, H-2′), 6.68 (d, *J* = 8.1 Hz, 1 H, H-5), 6.60 (d, *J* = 1.9 Hz, 1 H, H-2), 6.37 (s, 1 H, H-5′), 6.36 (dd, *J* = 8.1, 1.9 Hz, 1 H, H-6), 4.38 (dd, *J* = 2.7, 1.7 Hz, 1 H, H-8), 4.07 (d, *J* = 2.7 Hz, 1 H, H-7), 3.85 (s, 3 H, OMe), 3.75 (s, 3 H, OMe), 3.17 (dd, *J* = 16.6, 11.8 Hz, 1 H, H-7′a), 2.89 (dd, *J* = 16.6, 5.8 Hz, 1 H, H-7′b), 2.76 (ddd, *J* = 11.8, 5.8, 1.7 Hz, 1 H, H-8′).

^13^C-NMR (125 MHz, MeOD): δ (ppm) = 177.9, 148.9, 148.1, 146.1, 146.0, 137.4, 128.8, 127.8, 122.6, 118.1, 116.0, 113.7, 112.3, 74.4, 56.3, 56.3, 53.6, 40.8, 26.8. 

#### 4.3.7. Products **4B**

Isolated yield of two stereoisomers of **4B** was 19 mg (9%). Clear oil.

##### Major Isomer of Product **4B**:

^1^H-NMR (500 MHz, CDCl_3_): δ (ppm) = 6.86 (d, *J* = 8.1 Hz, 1 H H-5), 6.82 (dd, *J* = 8.1, 1.7 Hz, 1 H, H-6), 6.67 (d, *J* = 1.7 Hz, 1 H, H-2), 6.58 (s, 1 H, H-2′), 6.21 (s, 1 H, H-5′), 4.12 (dd, *J* = 10.5, 9.7 Hz, 1 H, H-8), 3.90 (s, 3 H, OMe), 3.87 (d, *J* = 9.7 Hz, 1 H, H-7), 3.86 (s, 3 H, OMe), 3.82 (s, 3 H, OMe), 3.78 (s, 3 H, COOMe), 3.59 (s, 3 H, OMe), 3.17 (dd, *J* = 16.2, 12.4 Hz, 1 H, H-7′a), 3.10 (dd, *J* = 16.2, 5.2 Hz, 1 H, H-7′b), 2.99 (ddd, *J* = 12.4, 10.5, 5.2 Hz, 1 H, H-8′).

^13^C-NMR (125 MHz, CDCl_3_): δ (ppm) = 175.0 (9′), 149.3 (3), 148.3 (4), 147.8 (4′), 147.7 (3′), 134.9 (1), 129.8 (6′), 126.1 (1′), 122.3 (6), 112.4 (5′), 112.1 (2), 111.3 (5), 110.5 (2′), 74.6 (8), 56.0 (OMe), 56.0 (OMe), 56.0 (OMe), 56.0 (OMe), 53.9 (7), 52.2 (COOMe) 47.6 (8′), 32.0 (7′).

HRMS: found 425.1584 (M^+^Na). C_22_H_26_O_7_Na^+^ requires 425.1571.

## Figures and Tables

**Table 1:** Conversions and ratio of product for all reactions. Compounds A, B, C and D are shown in Figure 6.

Entry	Subs.	Pre-cat (mol%)	Oxidant (equiv.)	Temp. (°C)	Time (h)	Conv [%]	Distribution of Products *				
							A	B	C	D	Others ^a^
**1**	**1**	**-**	**TBHP (10)**	**80**	**24**	-	-	-	-	-	-
**2**	**1**	[MoO_2_(SAP)]_2_ (0.5)	TBHP (5)	80	3	100	37	4	2	-	57
**3**	**1**	[MoO_2_(SATP)]_2_ (0.5)	TBHP (5)	80	3	100	37	4	2	-	57
**4**	**1**	-	mCPBA (1)	25	0.5	100	74	20	-	-	6
**5**	**2**	[MoO_2_(SAP)]_2_ (0.5)	TBHP (5)	80	8	100	29	18	5	-	48
**6**	**2**	[MoO_2_(SATP)]_2_ (0.5)	TBHP (5)	80	3.66	100	37	26	7	-	30
**7**	**2**	-	mCPBA (1)	25	0.33	100	48	52	-	-	-
**8**	**3**	[MoO_2_(SAP)]_2_ (2.5)	TBHP (2)	80	1.66	0	-	-	-	-	-
**9**	**3**	[MoO_2_(SAP)]_2_ (1.5)	TBHP (5)	80	2.5	100	1	1	-	-	98
**10**	**3**	[MoO_2_(SATP)]_2_ (2.5)	TBHP (2)	80	1.5	0	-	-	-	-	-
**11**	**3**	[MoO_2_(SATP)]_2_ (0.5)	TBHP (5)	80	20	99	1	1	-	-	97
**12**	**3**	-	mCPBA (1)	25	0.5	96	71	24	-	-	1
**13**	**4**	[MoO_2_(SAP)]_2_ (0.5)	TBHP (2)	80	24	0-	-	-	-	-	-
**14**	**4**	[MoO_2_(SAP)]_2_ (1.5)	TBHP (5)	80	21	93	-	14	2	-	84
**15**	**4**	[MoO_2_(SATP)]_2_ (1.5)	TBHP (5)	80	2	80	-	19	4	-	77
**16**	**4**	-	mCPBA (1)	25	0.5	83	-	32	-	51	-

Substrate concentration: 50 mM substrate in toluene (or DCM for *m*CPBA reactions); oxidant: TBHP (70 wt% in H_2_O); *m*CPBA (70 wt% in H_2_O and mCBA); * ratios are calculated from integral values in ^1^H-NMR spectra of reaction mixtures; ^a^ non-characterized mono- and oligomers.

## Supplementary Materials

The following are available online, ^1^H-NMR, ^13^C-NMR and HSQC spectra of the major isomers or diastereomeric mixtures of **1A**, **1B, 2A**, **2B, 3A**, **3B,** and **4B.**
