# Supplementary material for: Formation of Tetrahydrofurano-, Aryltetralin, and Butyrolactone Norlignans through the Epoxidation of 9-Norlignans
Source: Molecules. 2020 Mar 5;25(5):1160. doi: 10.3390/molecules25051160 (PMC7179189; doi:10.3390/molecules25051160)

Supplementary Materials

Formation of Tetrahydrofurano-, Aryltetralin, and Butyrolactone Norlignans through the Epoxidation of 9-Norlignans

Patrik A. Runeberg [1], Dominique Agustin [2,3] and Patrik C. Eklund [1,*]

[1] Laboratory of Molecular Science and Engineering, Åbo Akademi University, Biskopsgatan 8, 20500 Åbo, Finland; patrik.runeberg@abo.fi

[2] LCC-CNRS, Université de Toulouse, CNRS, UPS, 31077 Toulouse, France; dominique.agustin@iut-tlse3.fr

[3] Institut Universitaire de Technologie Paul Sabatier, Département de Chimie, Av. G. Pompidou, CS20258, F-81104 Castres, France

* Correspondence: paeklund@abo.fi; Tel.: + 358-2-215 4720

Table of Contents

^{1}H NMR, ^{13}C NMR and HSQC of: **1A**....................S2 - S4

1B....................S5 - S7

2A....................S8 - S10

2B....................S11 - S13

3A (isomer 1+3) ....S14 - S16

3A (isomer 2) ....S17 - S19

3A (isomer 4) ....S20 - S22

3B....................S23 - S25

4B....................S26 - S28

^{1}H NMR ($CDCl_3$) of two diastereomers of 1A (traces seen of two other diastereomers)

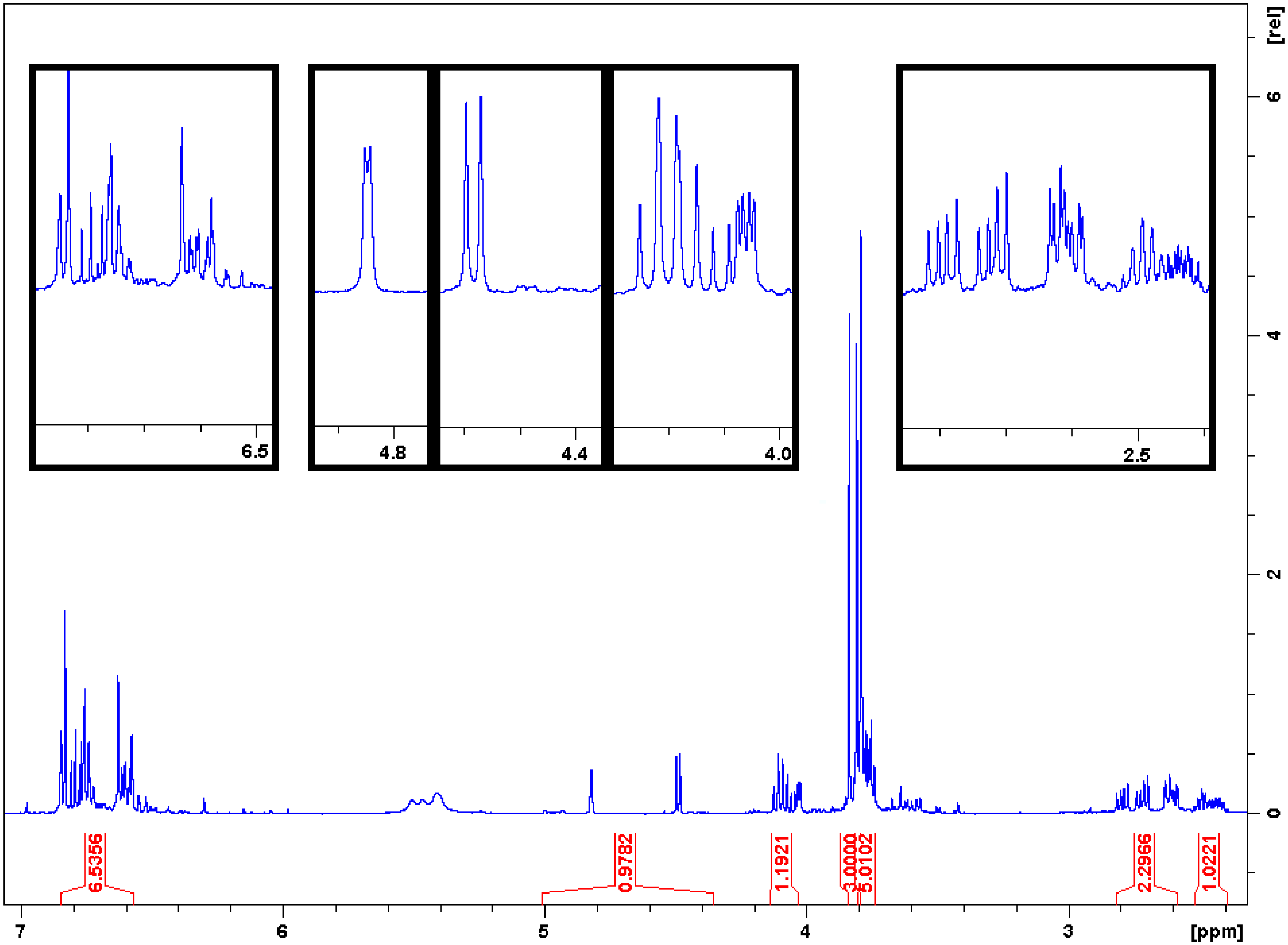

^{13}C NMR ($CDCl_3$) of two diastereomers of 1A

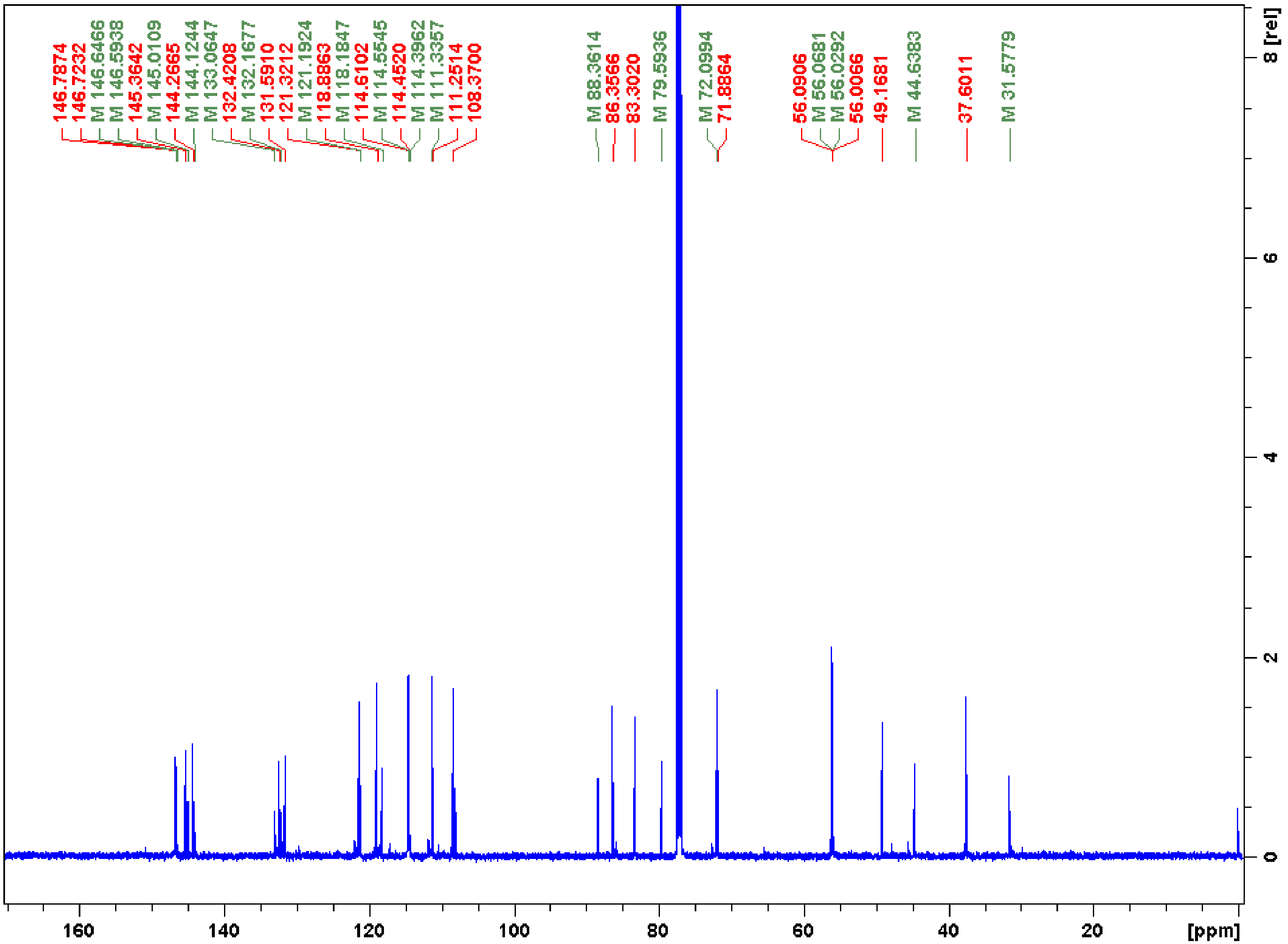

HSQC ($CDCl_3$) of diastereomeric mixture of 1A (signals solved for the major isomer

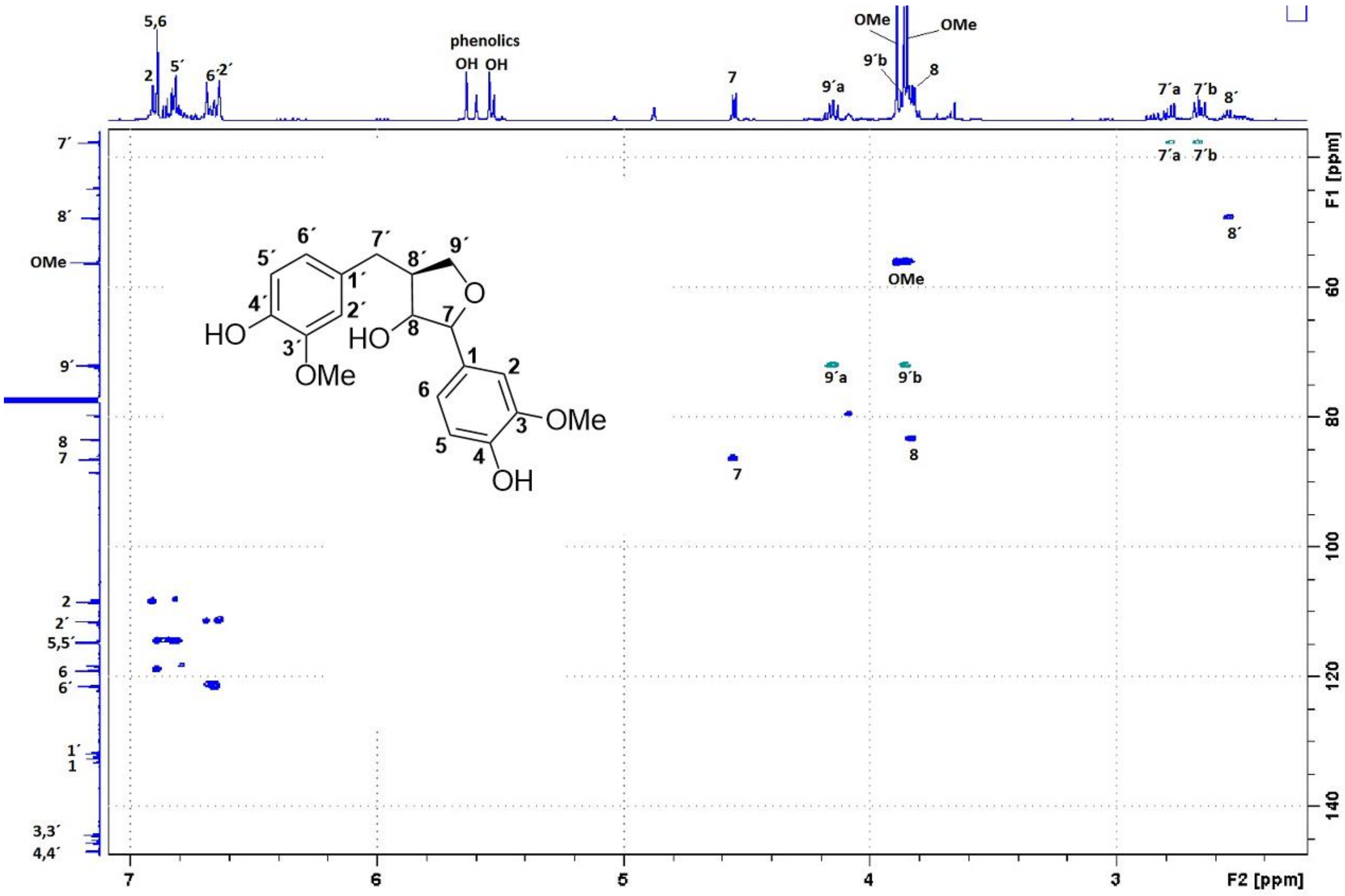

^{1}H NMR ($CDCl_3$) of 1B

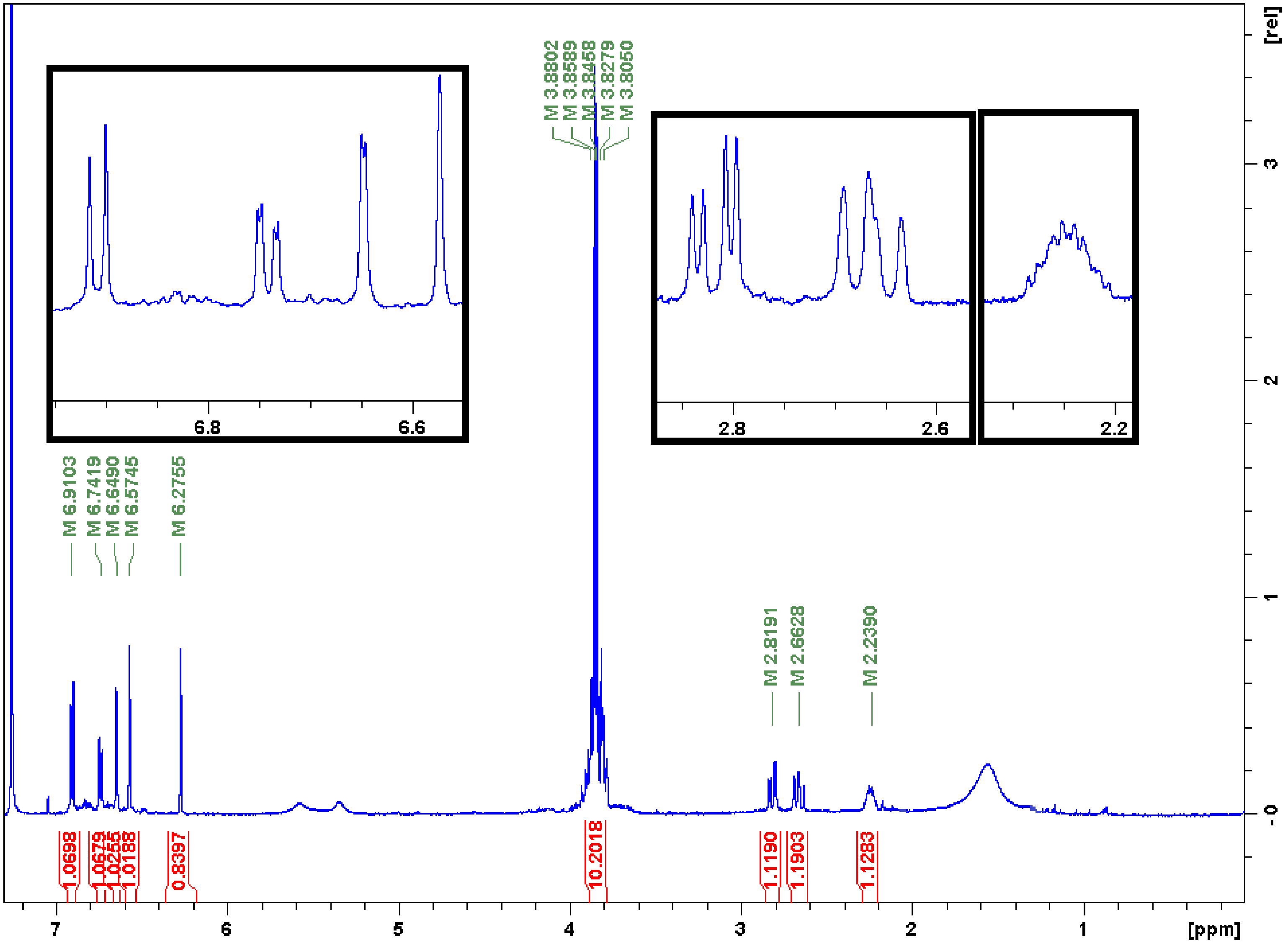

^{13}C NMR ($CDCl_3$) of 1B

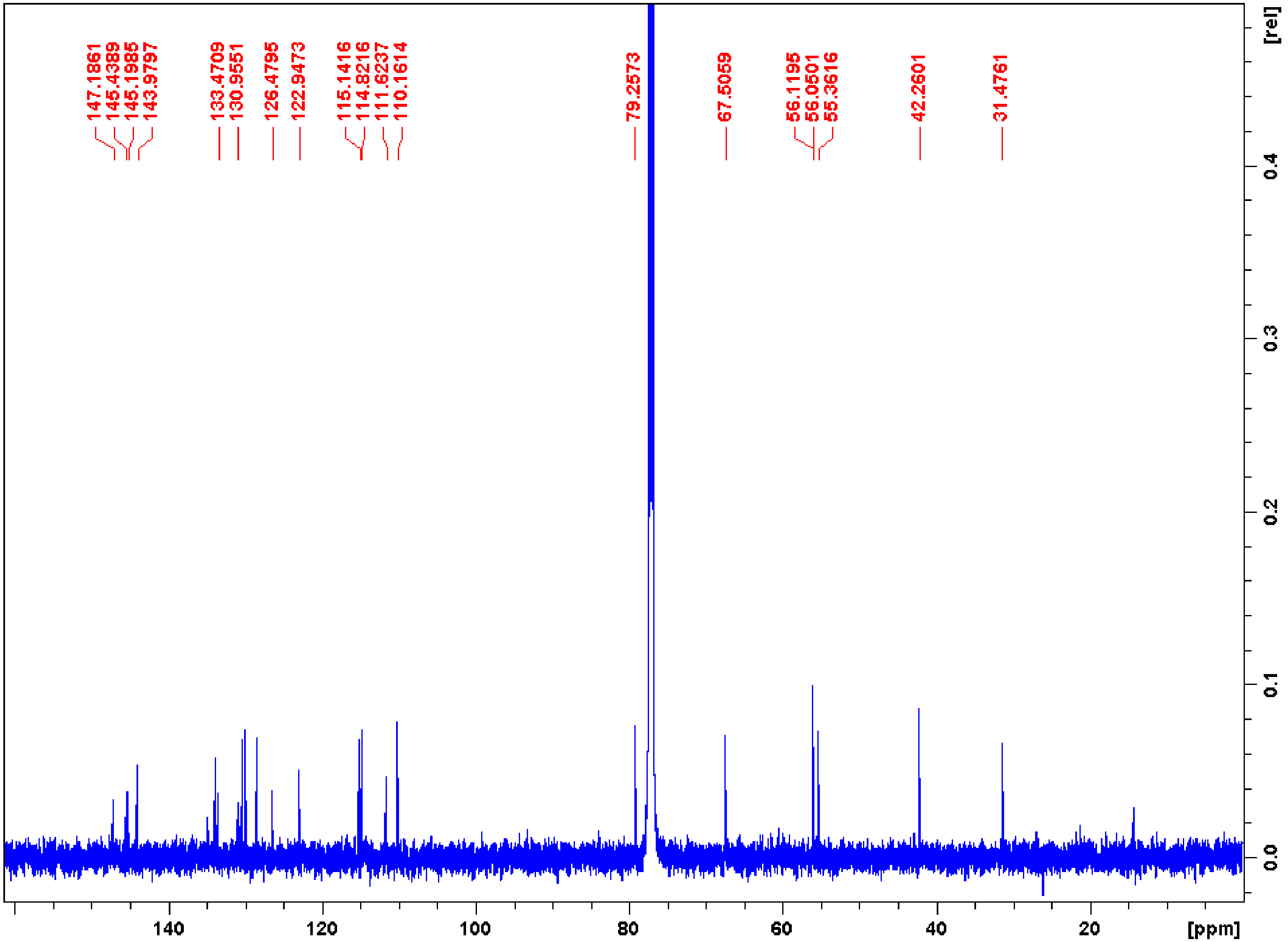

HSQC ($CDCl_3$) of 1B

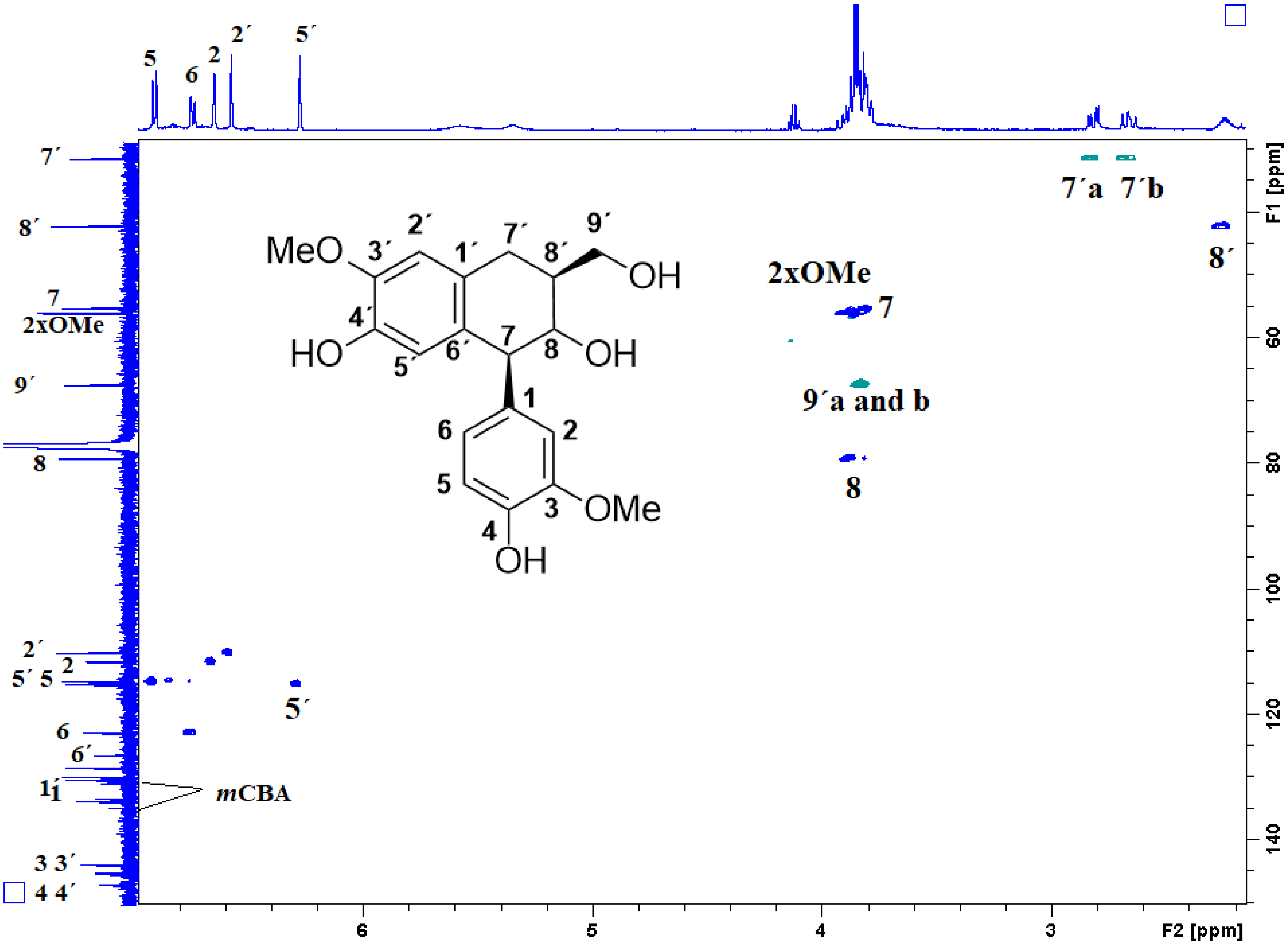

^{1}H NMR ($CDCl_3$) of major diastereomer of 2A (traces of 2 other diastereomers seen)

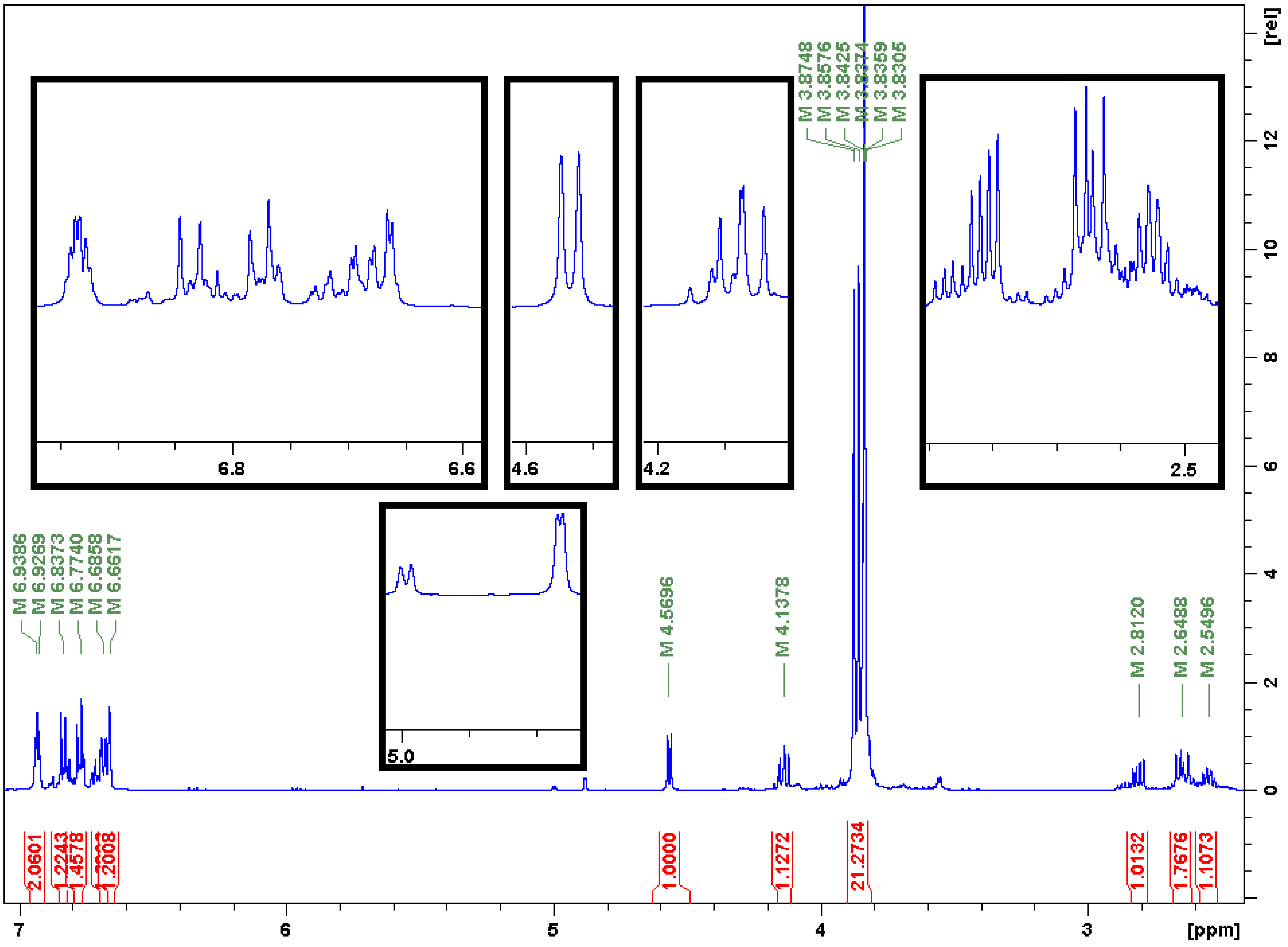

^{13}C NMR ($CDCl_3$) of major diastereomer of 2A (traces of 2 other diastereomers seen)

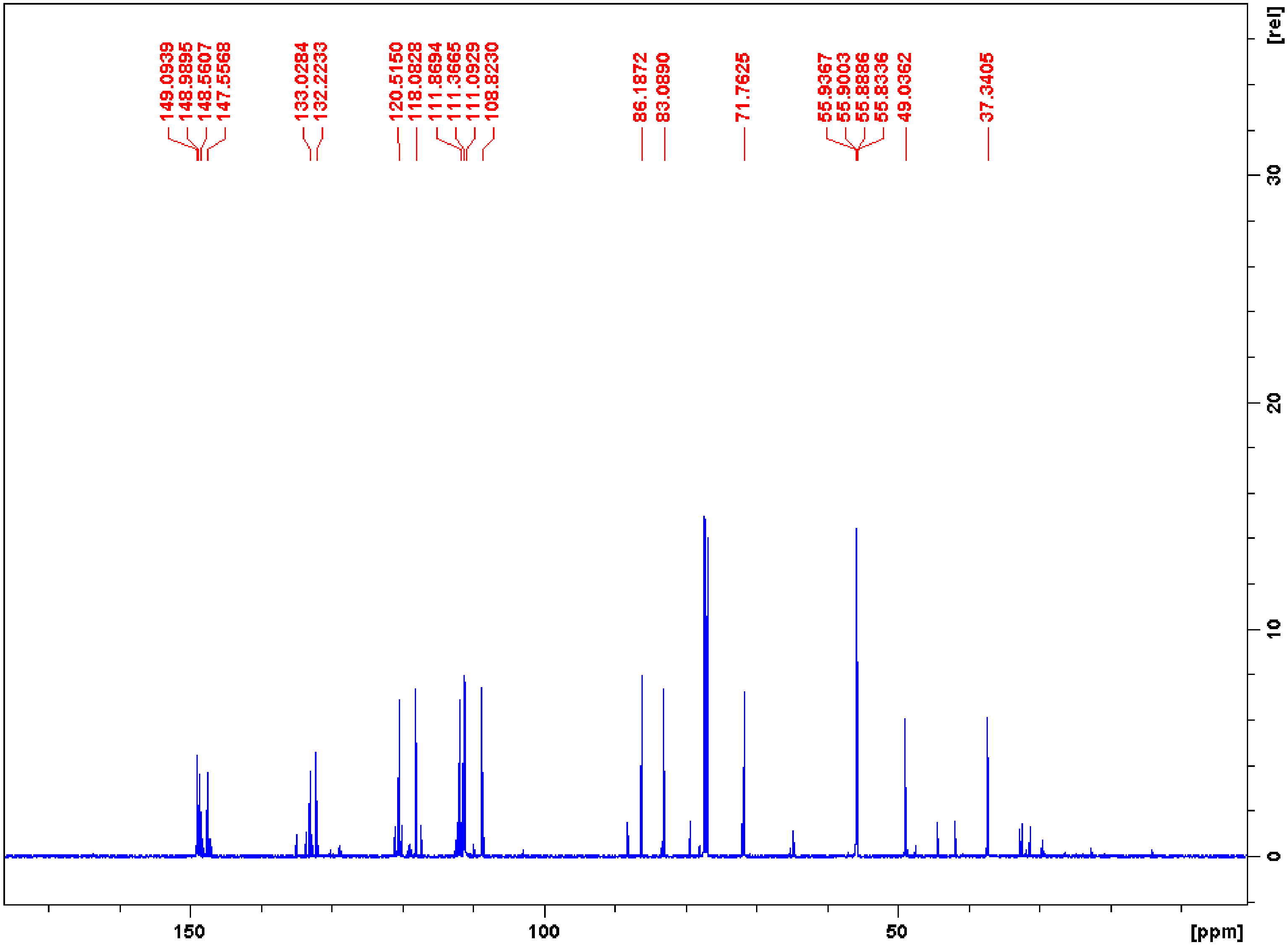

HSQC ($CDCl_3$) of 2A

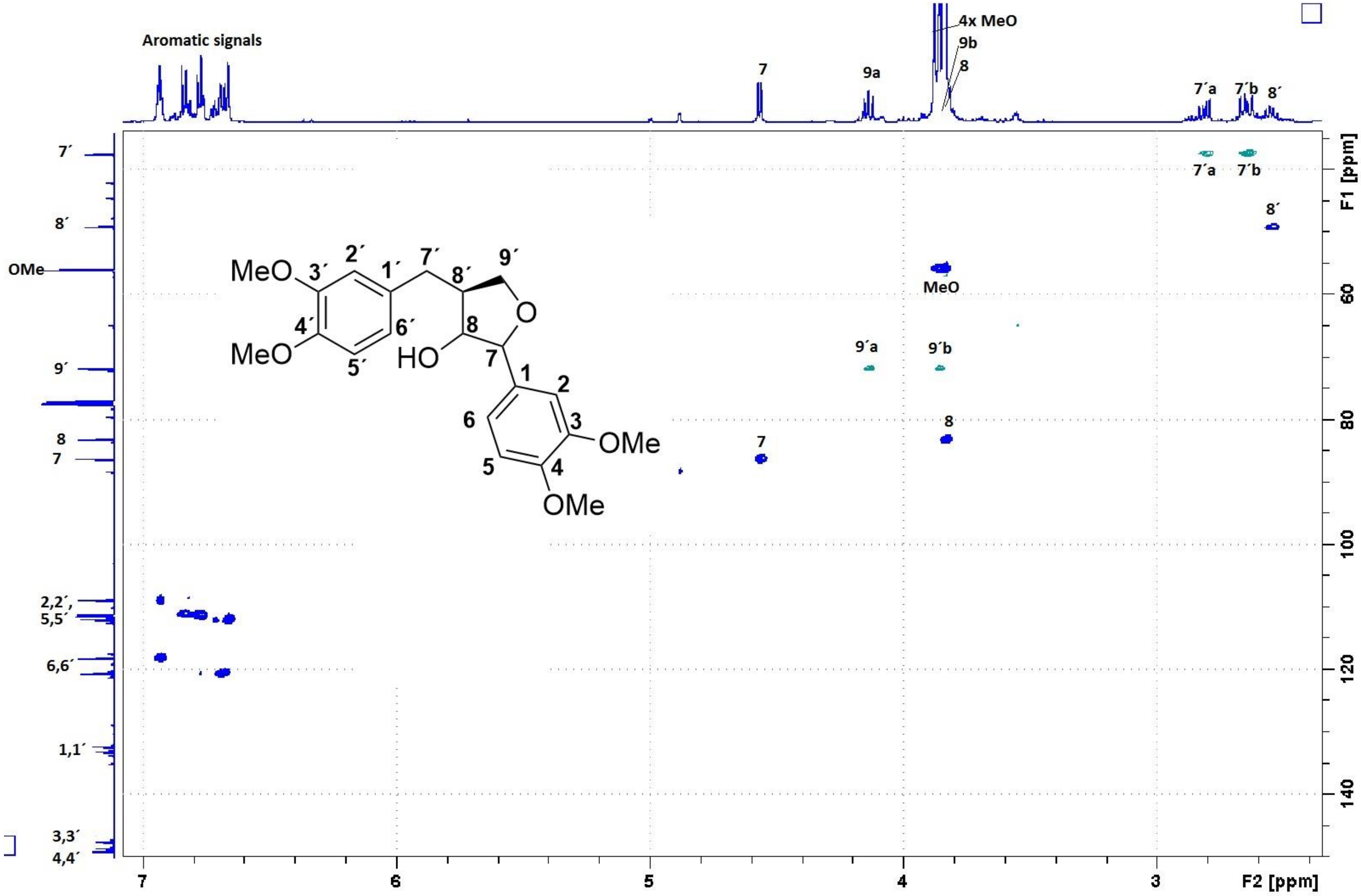

^{1}H NMR ($CDCl_3$) of 2B

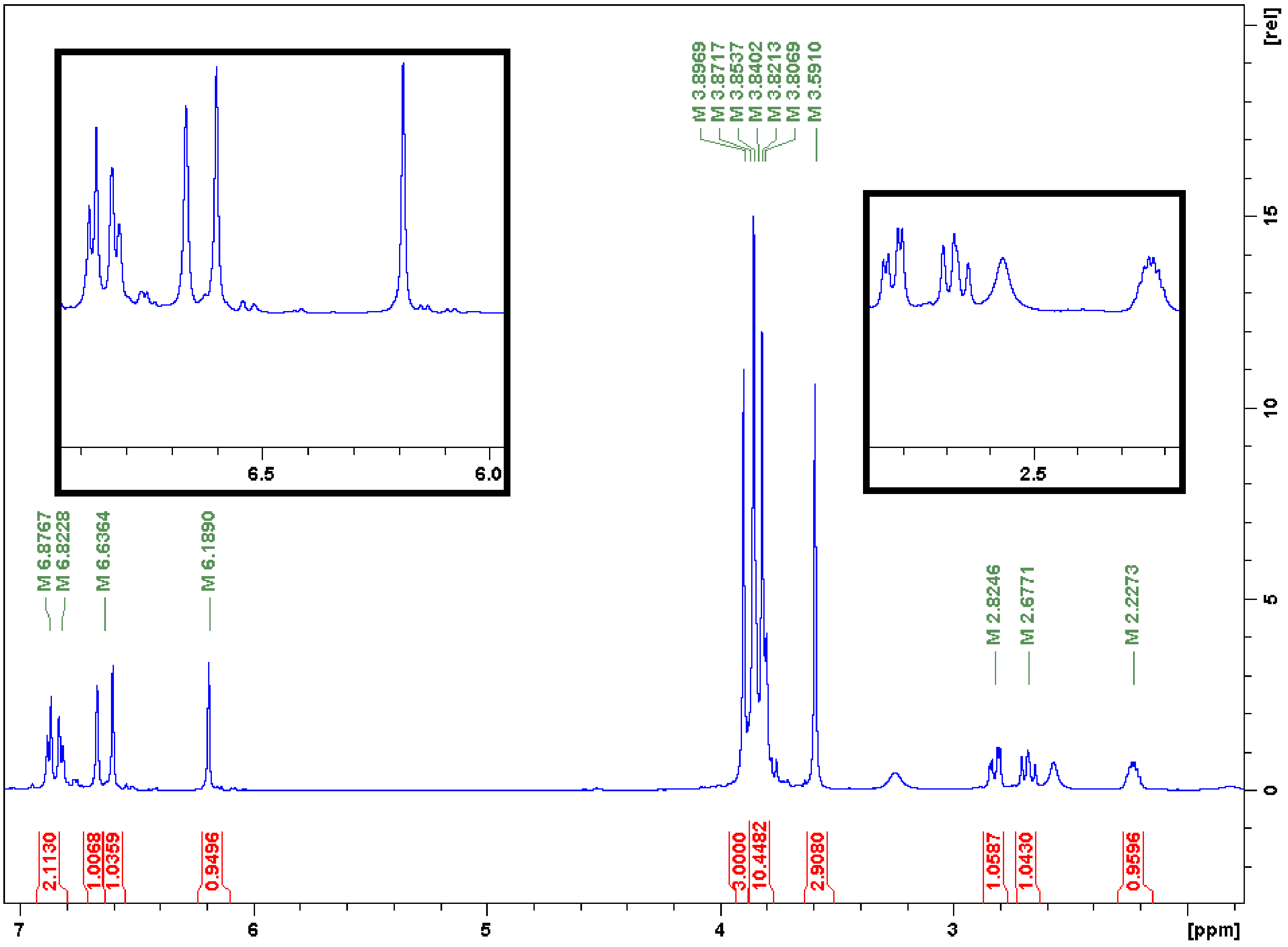

^{13}C NMR ($CDCl_3$) of 2B

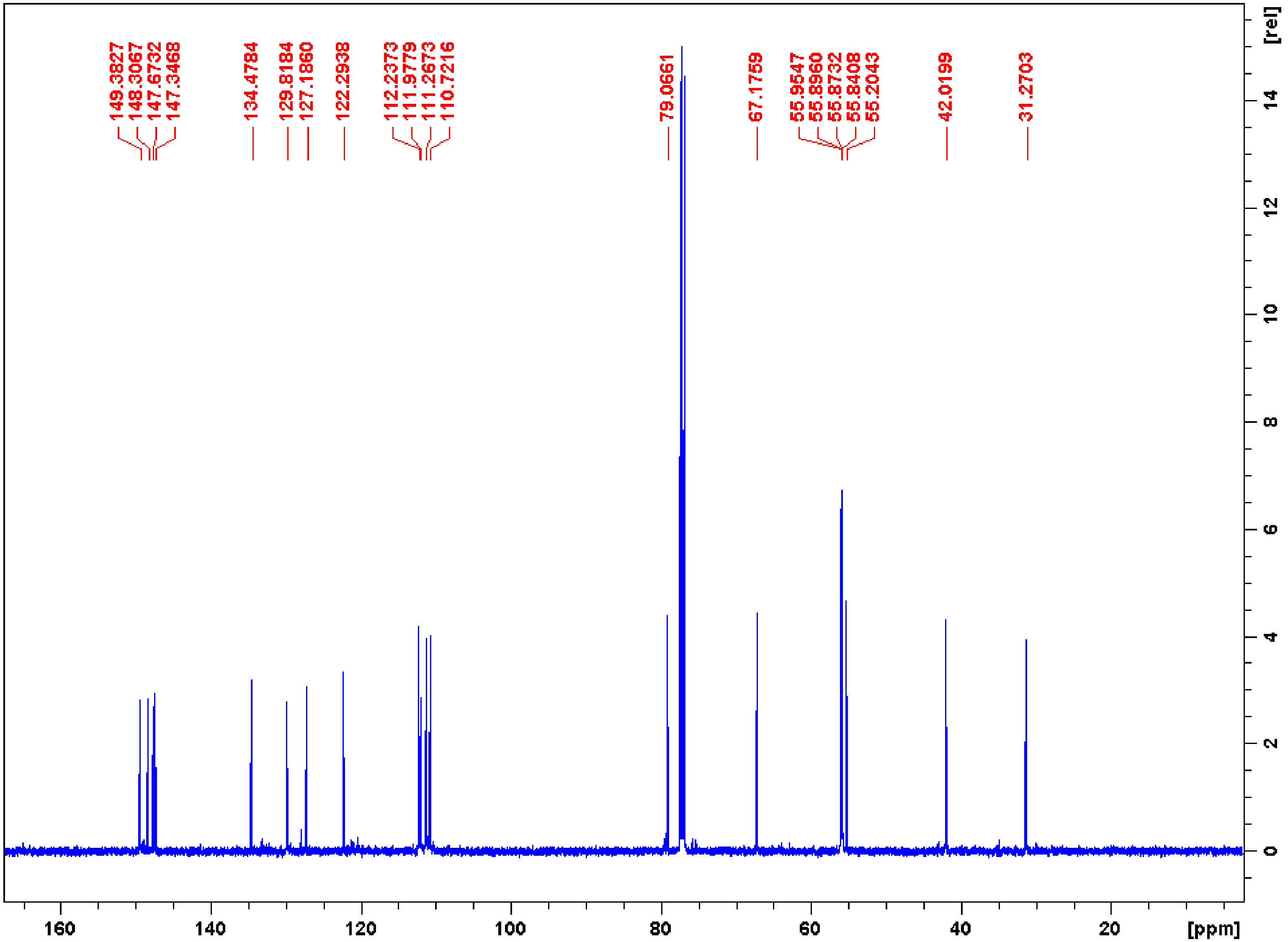

HSQC ($CDCl_3$) of 2B

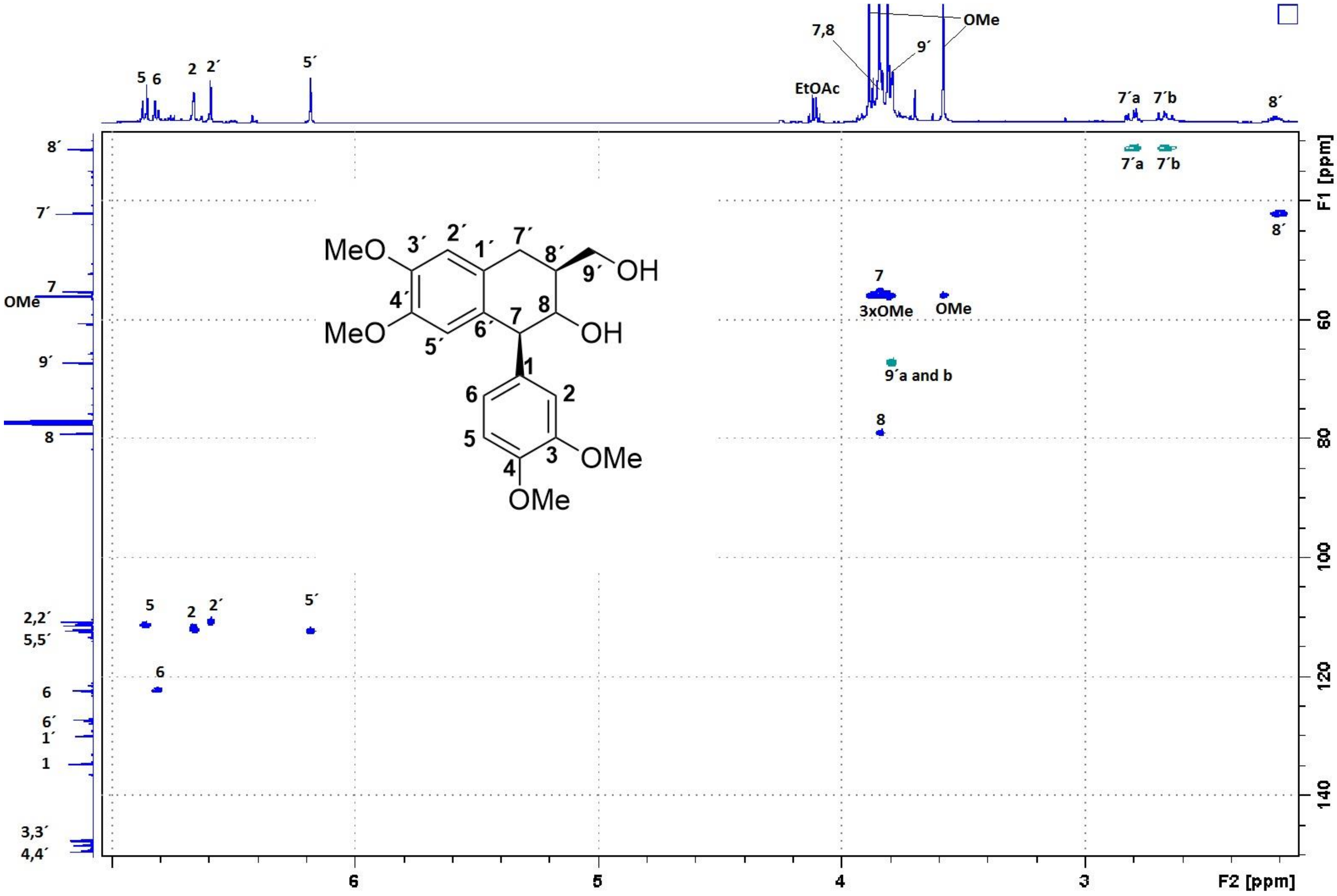

^{1}H NMR (MeOD) of isomer 1 of 3A (isomer 3 + traces of isomers 2 and 4 also seen)

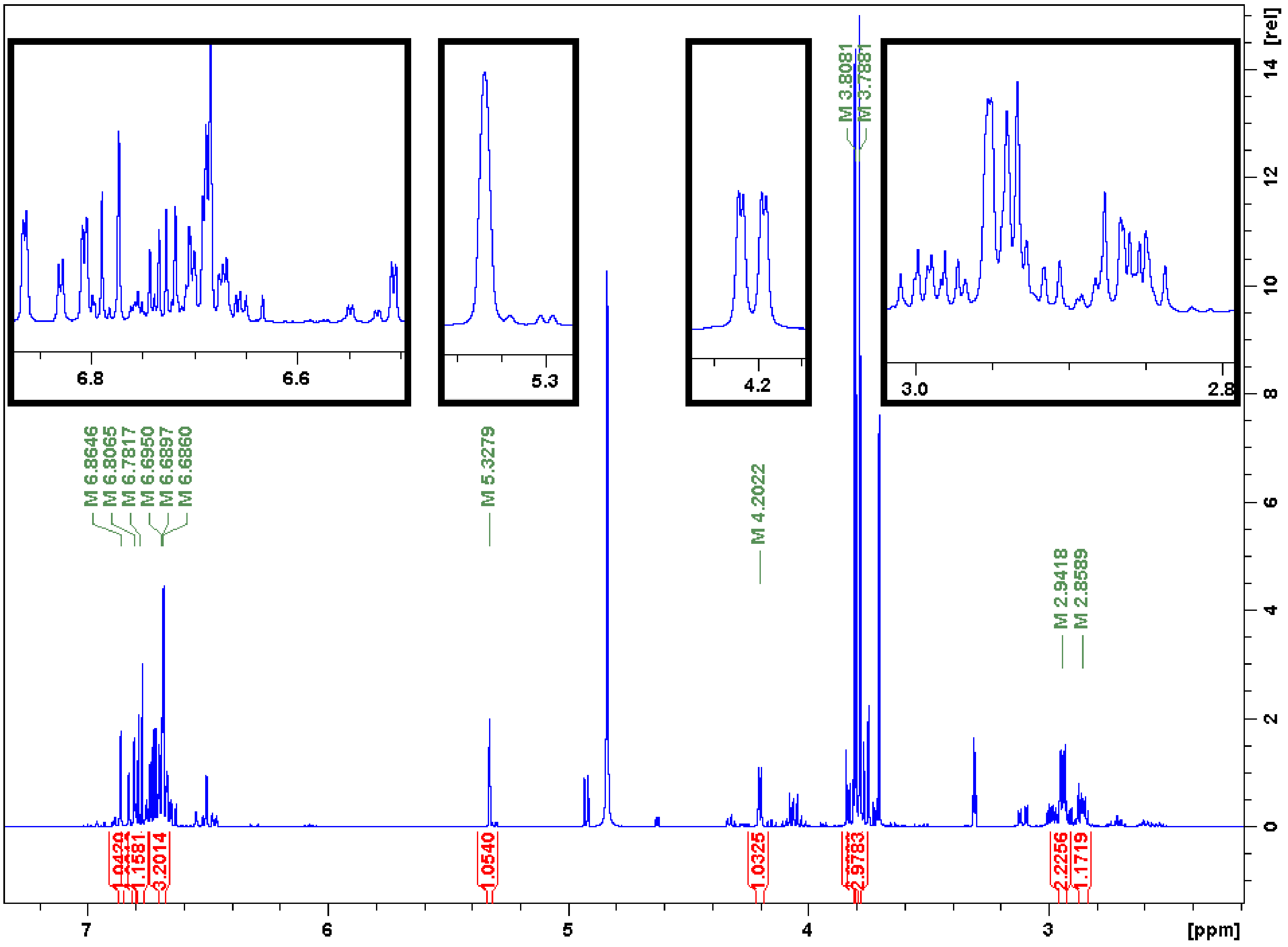

^{13}C NMR (MeOD) of isomer 1 and isomer 3 of 3A (traces of isomers 2 and 4 also seen)

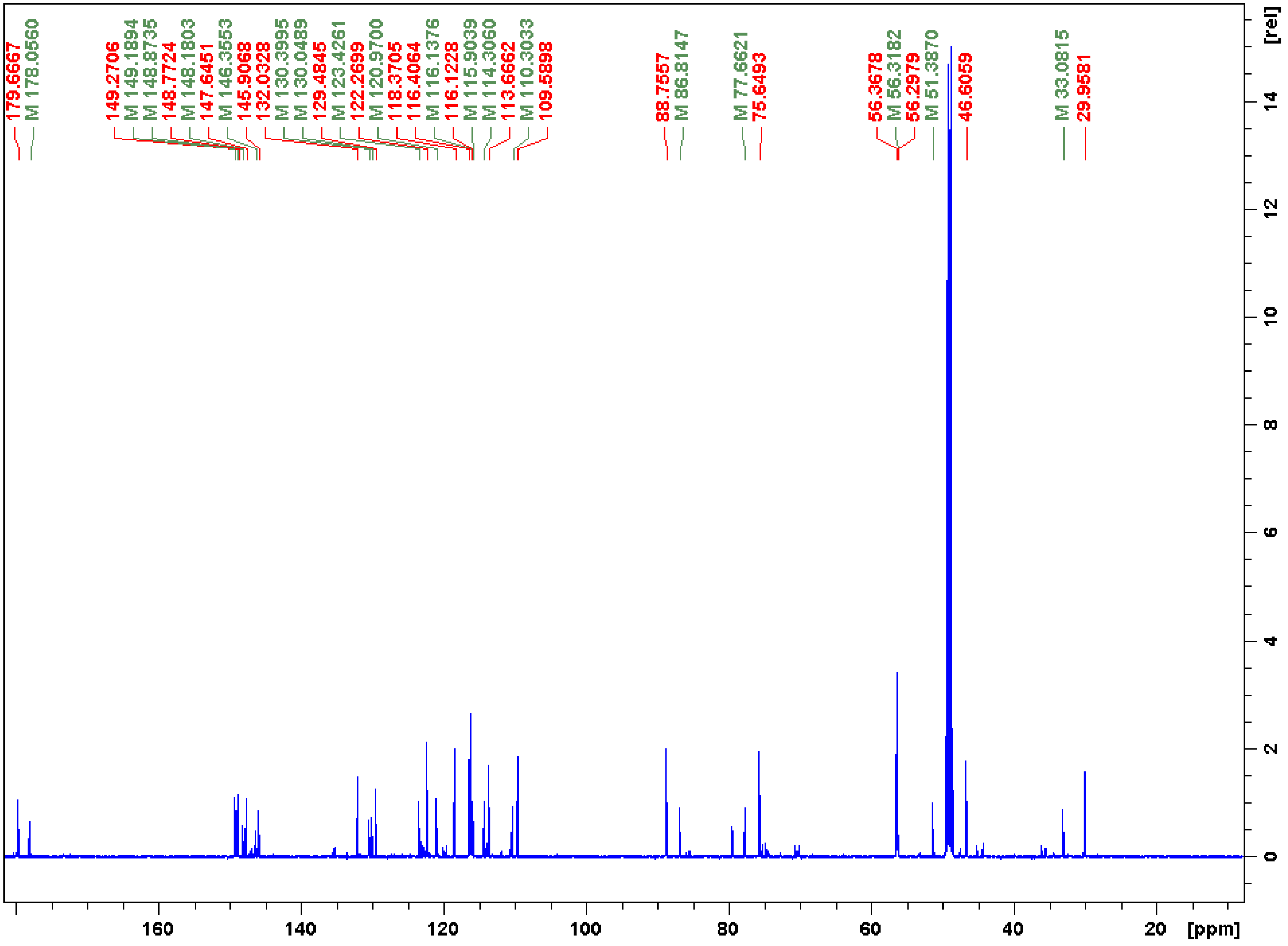

HSQC of isomer 1 of 3A (isomer 3 + traces of isomers 2 and 4 also seen)

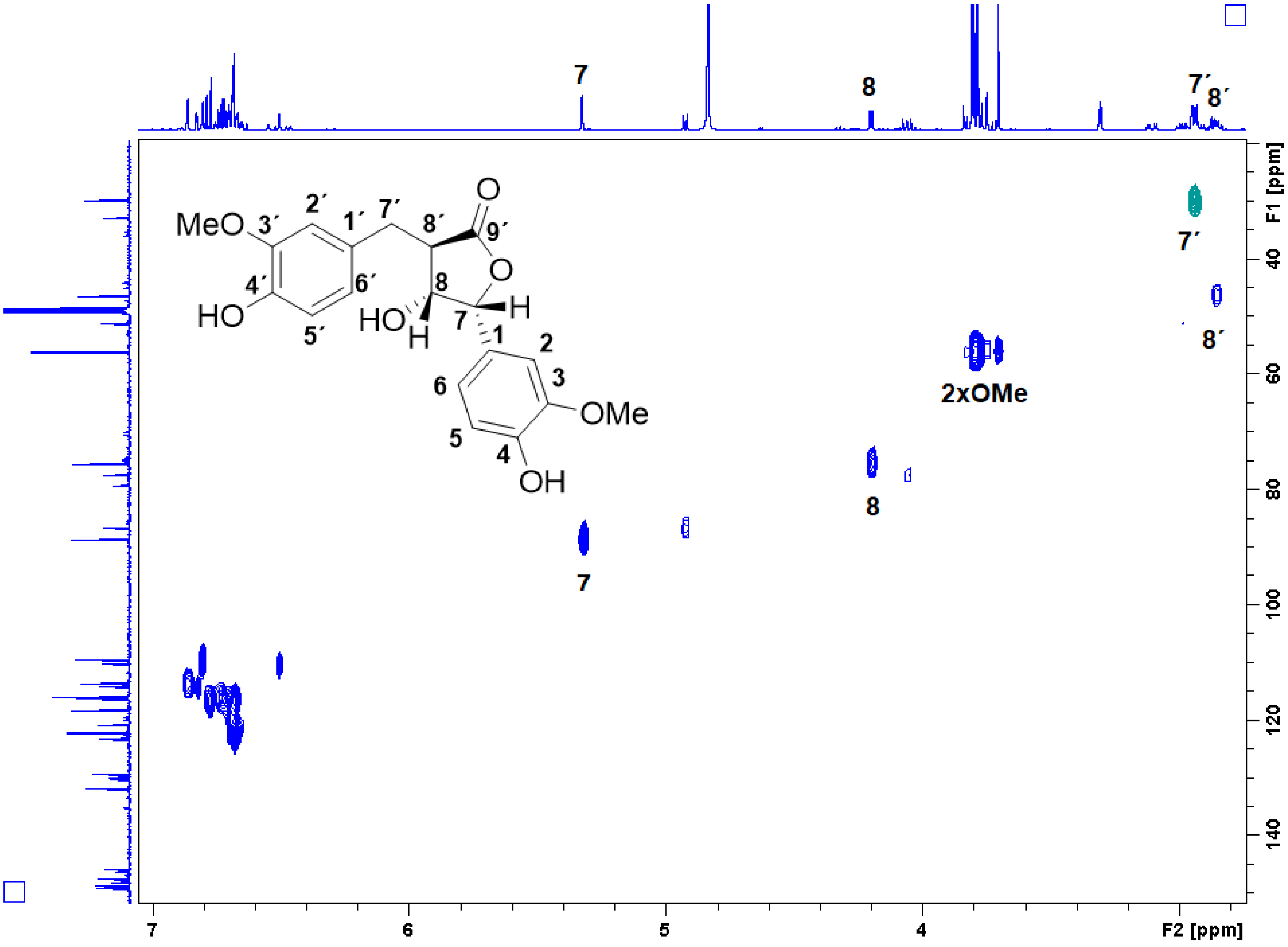

^{1}H NMR (MeOD) of isomer 2 of 3A

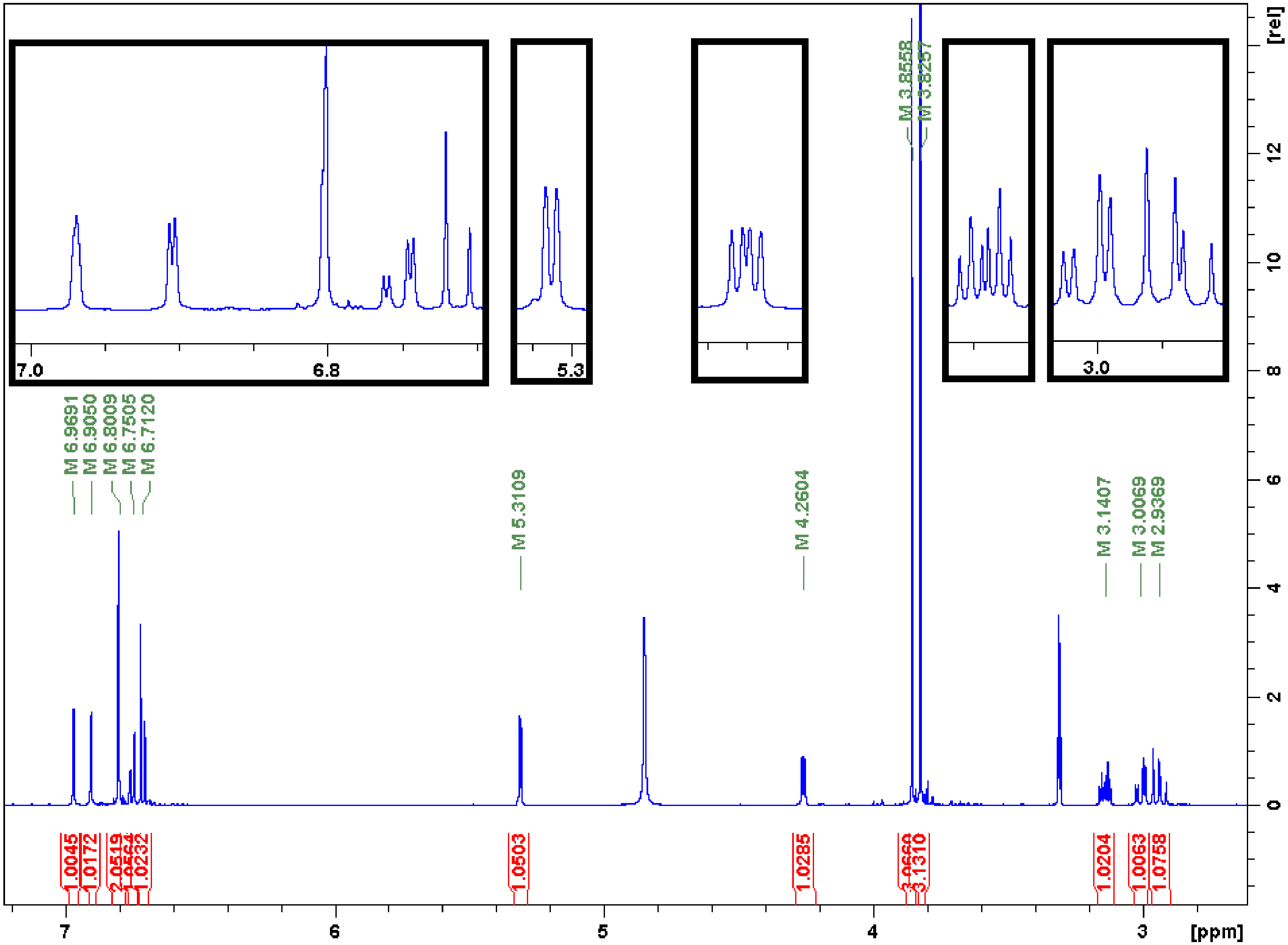

^{13}C NMR (MeOD) of isomer 2 of 3A

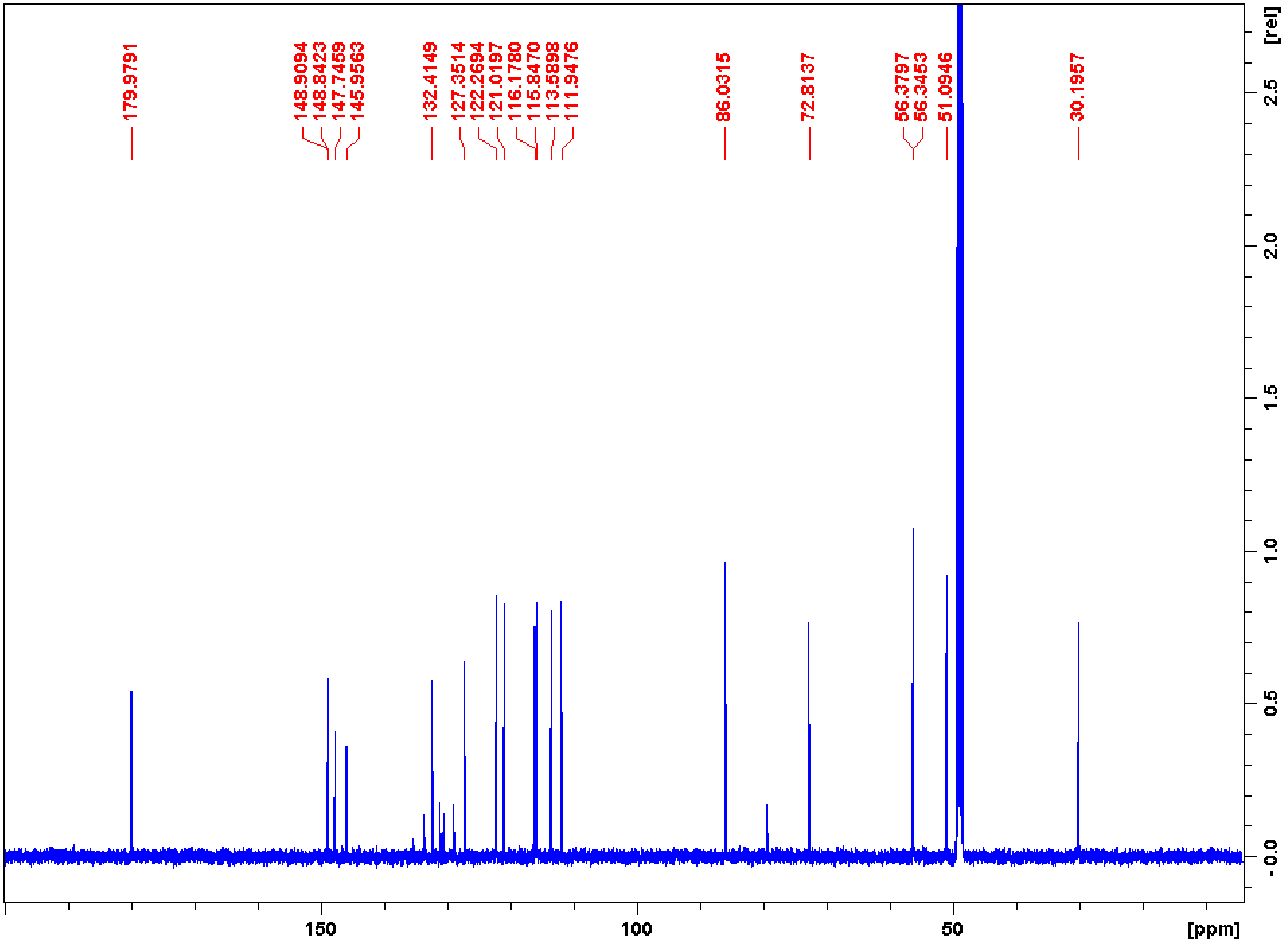

HSQC (MeOD) of isomer 2 of 3A

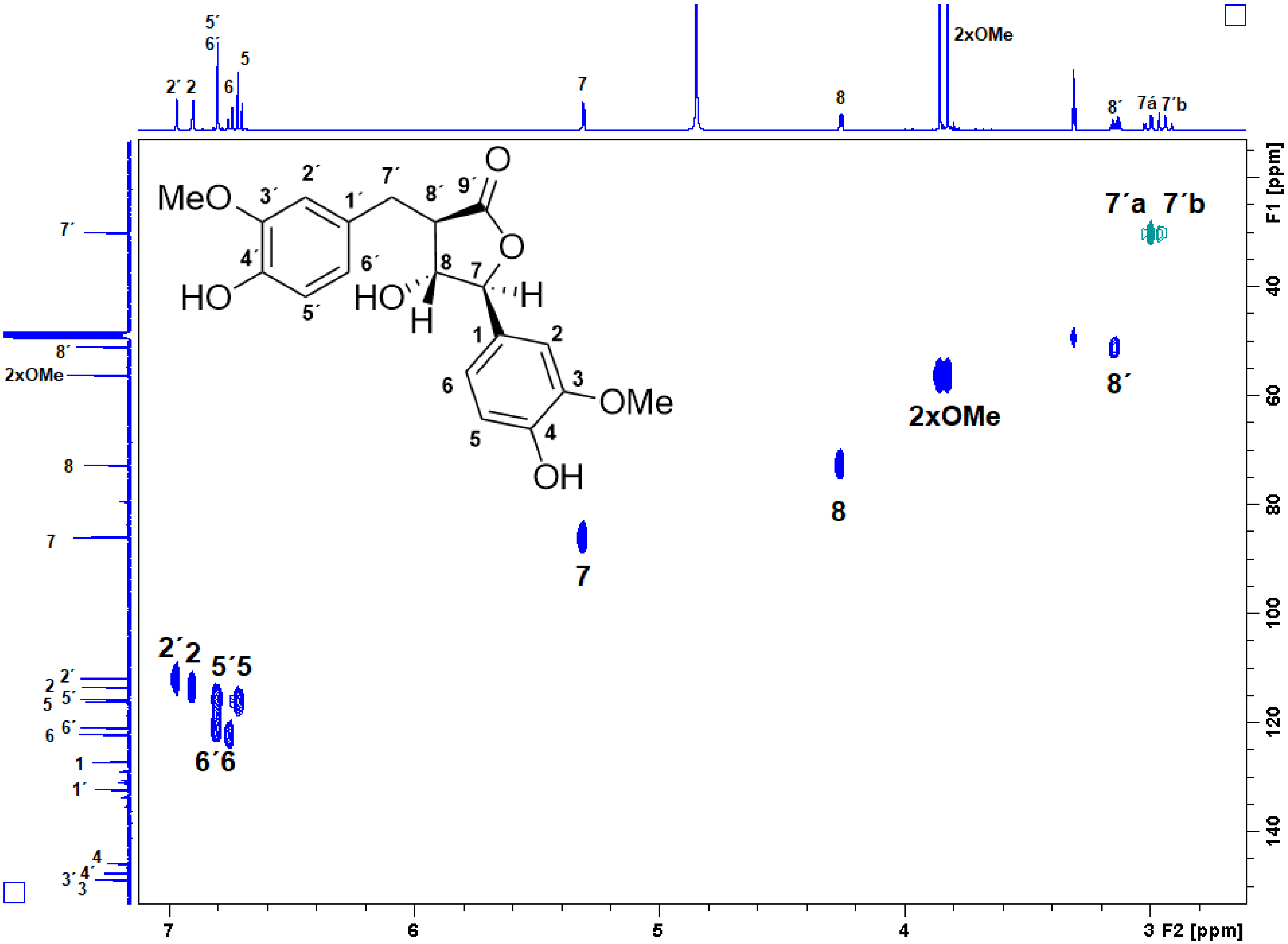

^{1}H NMR (MeOD) of isomer 4 of 3A (traces of other isomers)

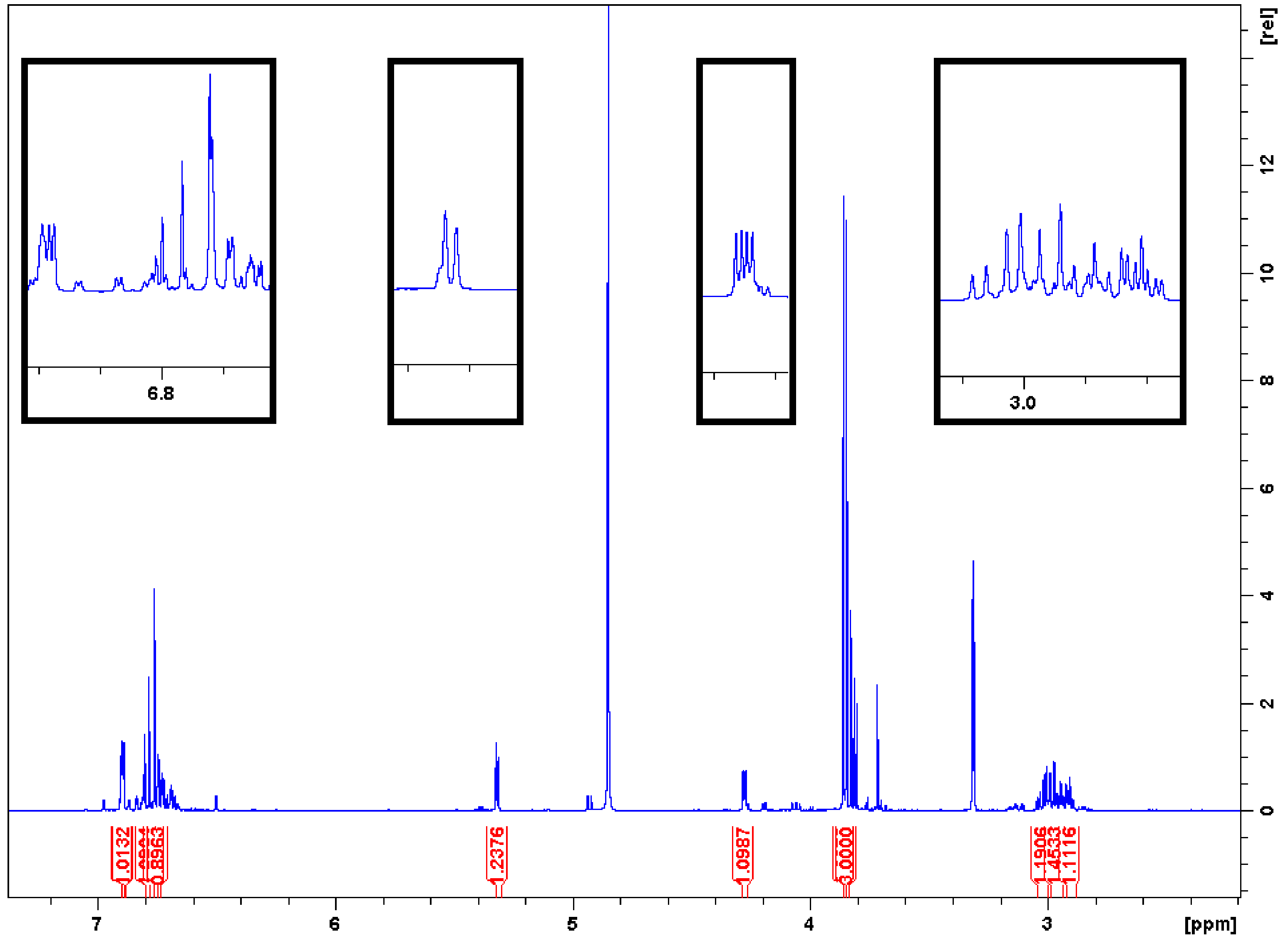

^{13}C NMR (MeOD) of isomer 4 of 3A (traces of other isomers)

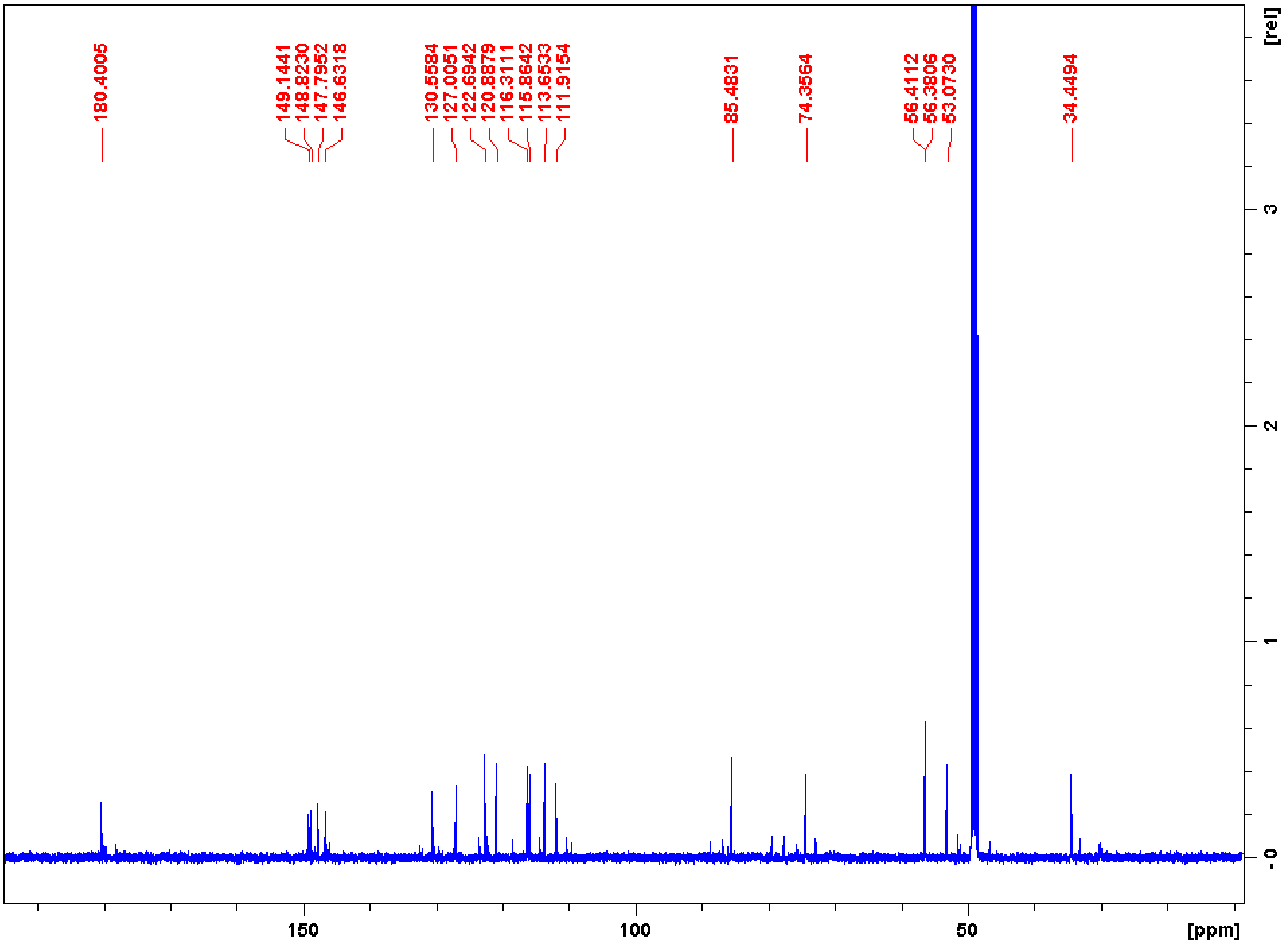

HSQC of isomer 4 of 3A (traces of other isomers)

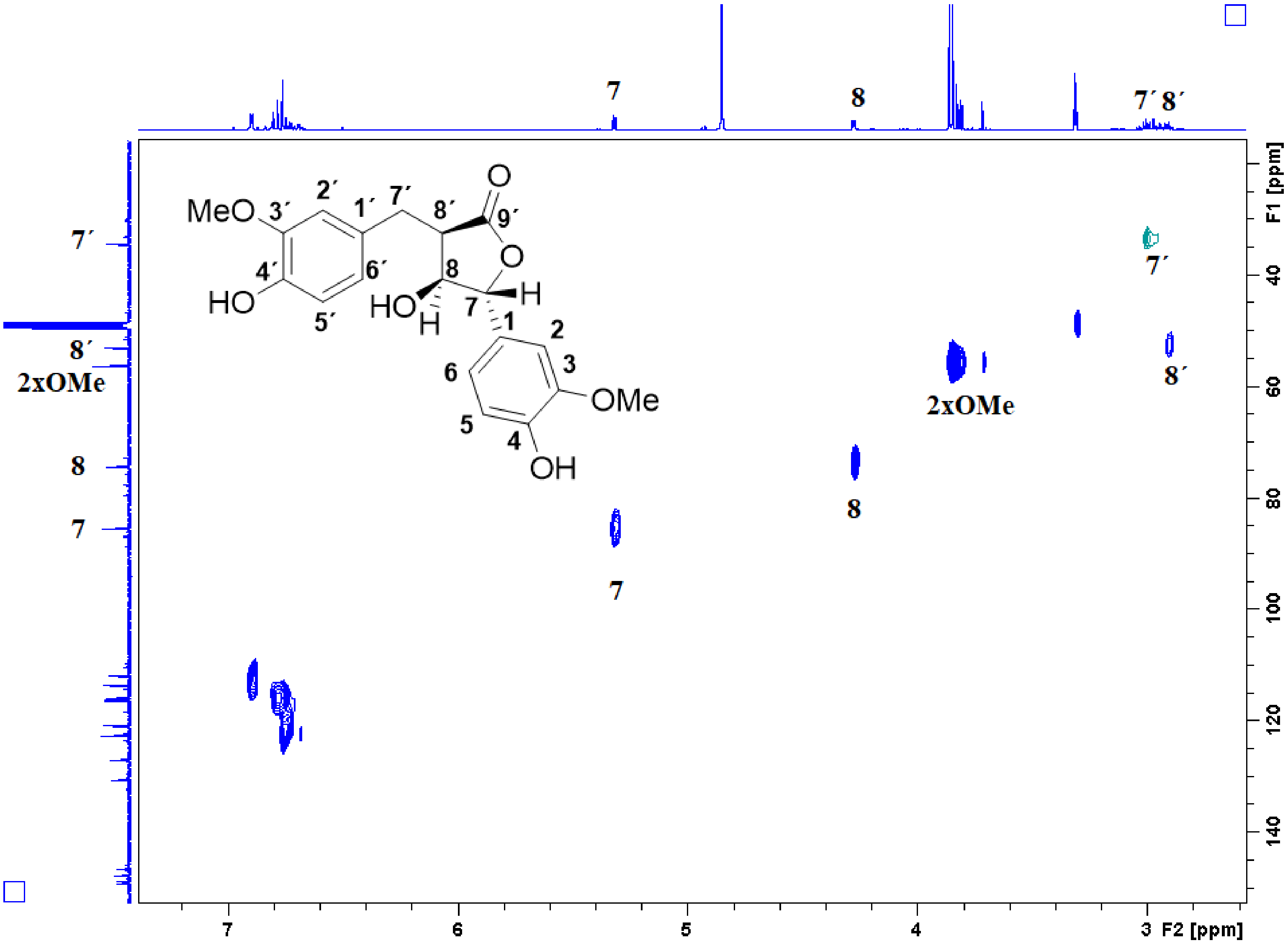

^{1}H NMR (MeOD) of isomer 1 and isomer 2 of 3B

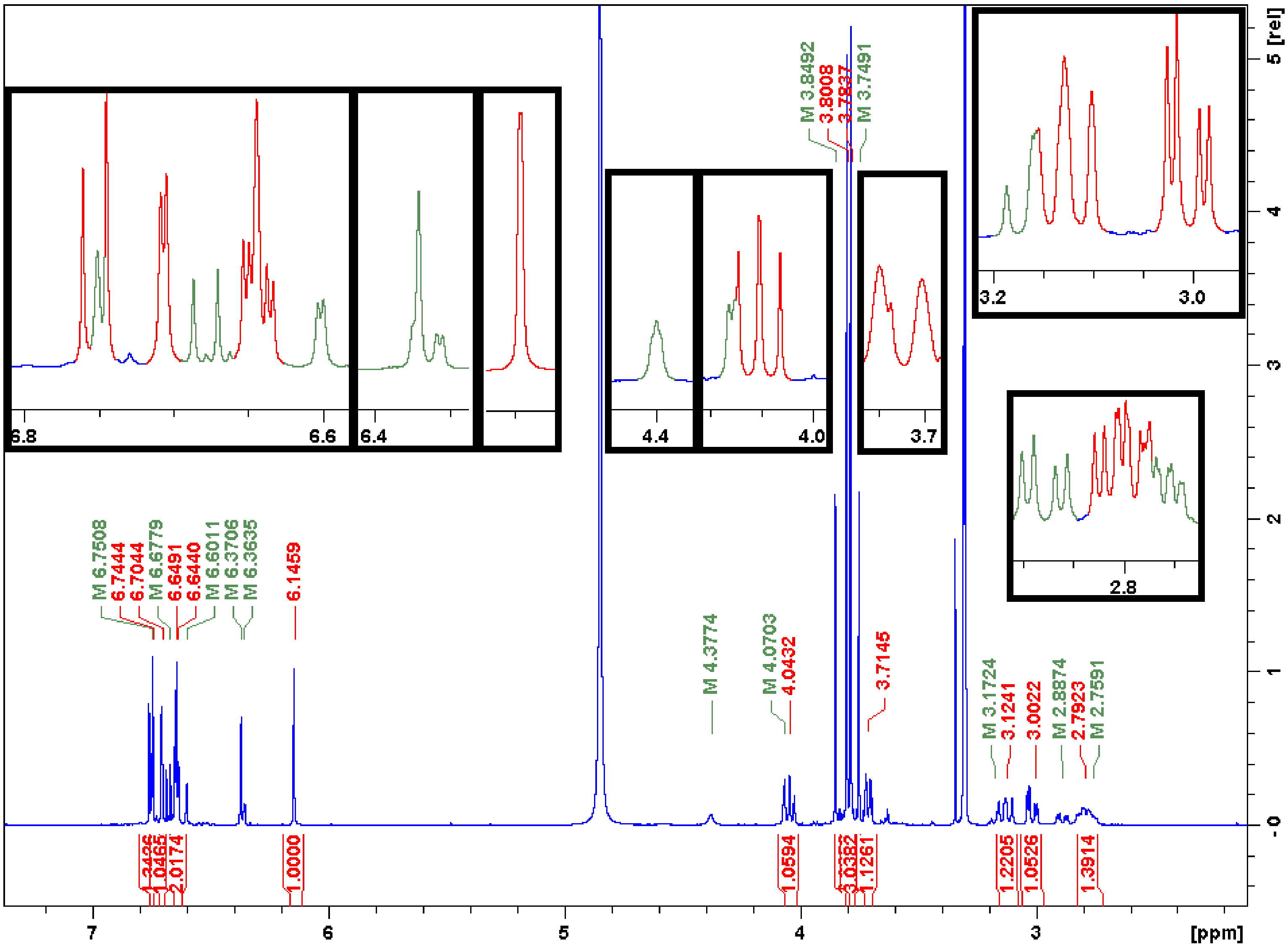

^{13}C NMR (MeOD) of isomer 1 and isomer 2 of 3B

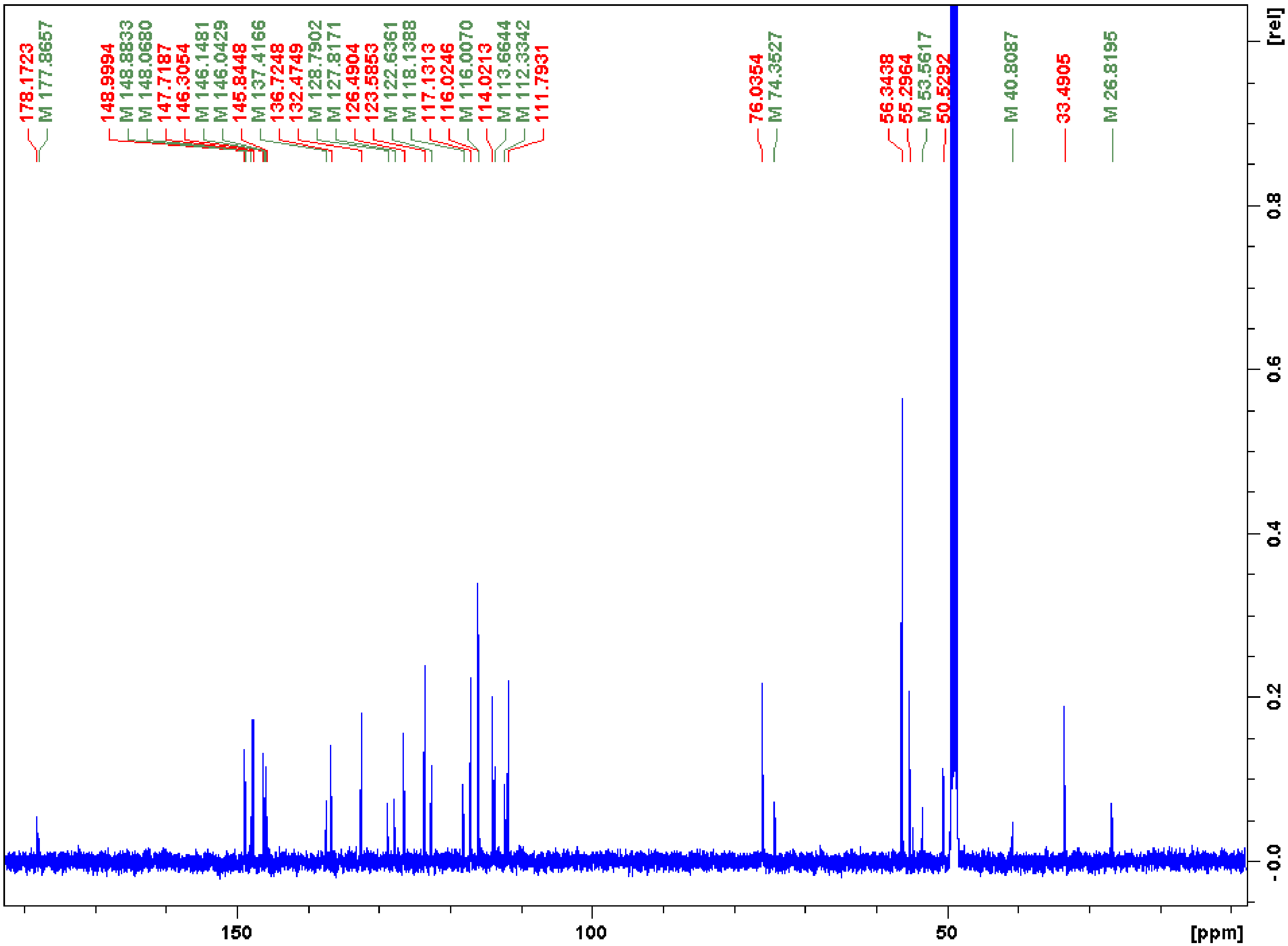

HSQC (MeOD) of isomer 1 and isomer 2 3B

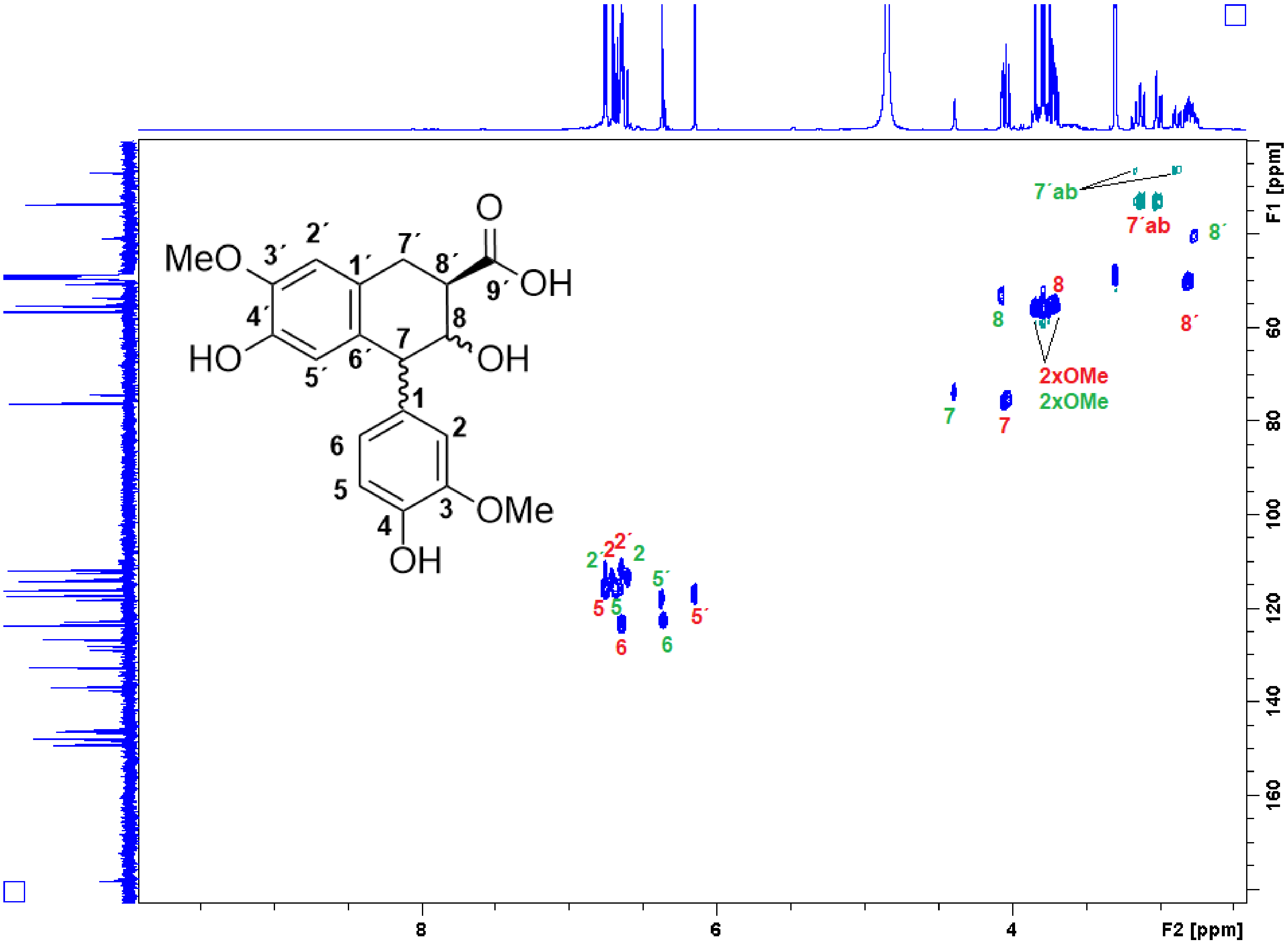

^{1}H NMR ($CDCl_3$) of 4B (traces of other diastereomers seen)

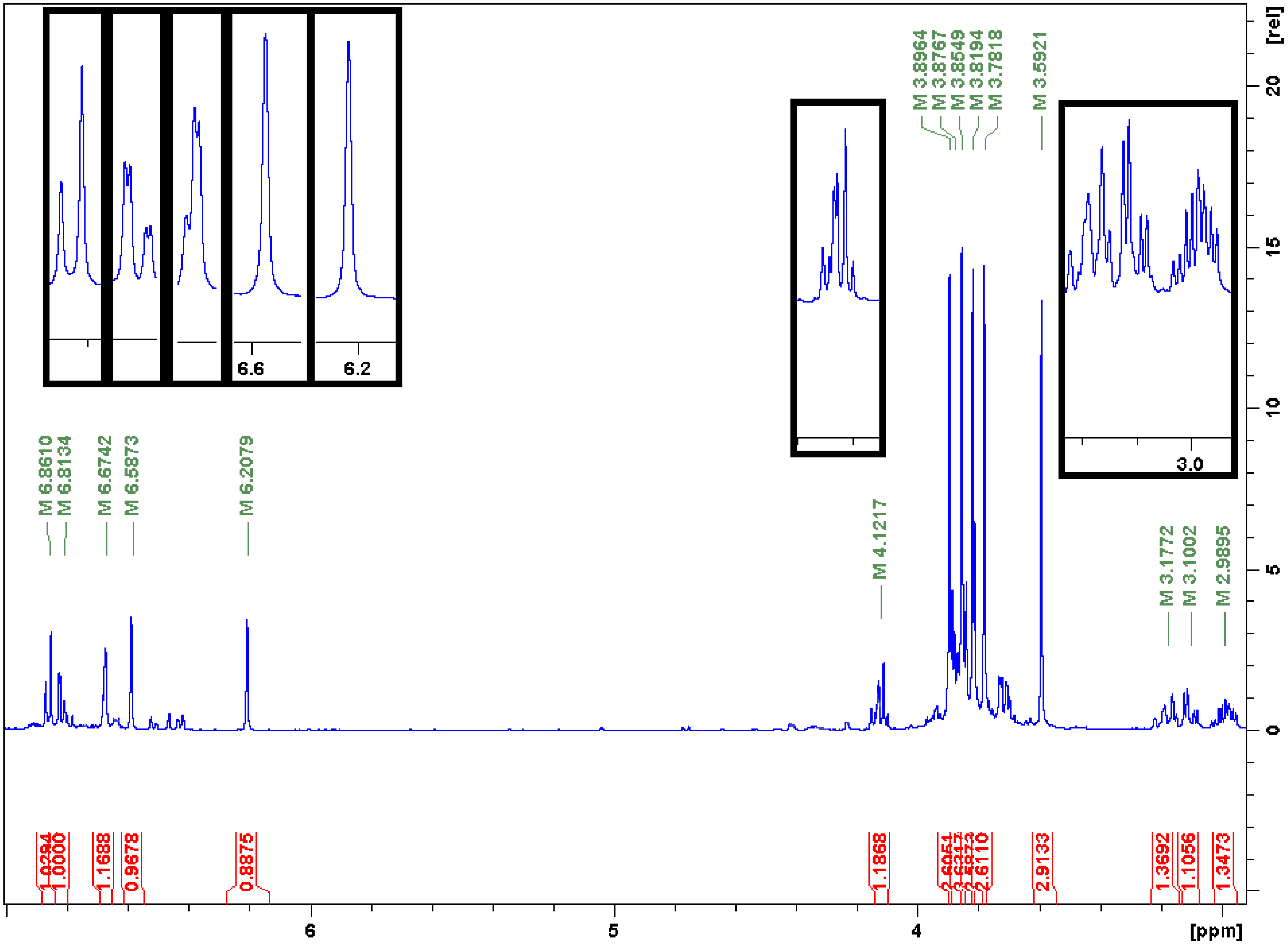

^{13}C NMR ($CDCl_3$) of 4B (traces of other diastereomers seen)

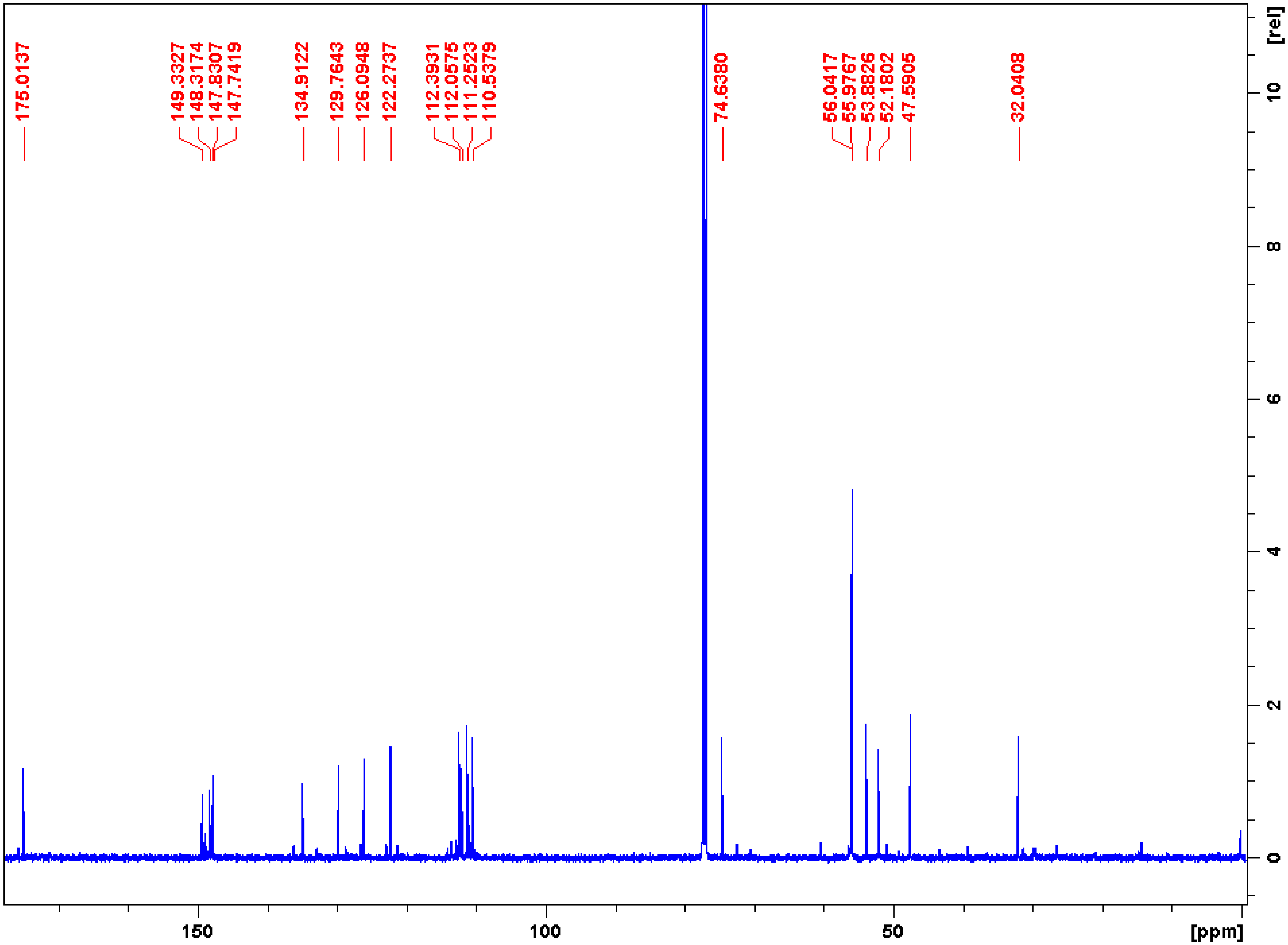

HSQC ($CDCl_3$) of 4B

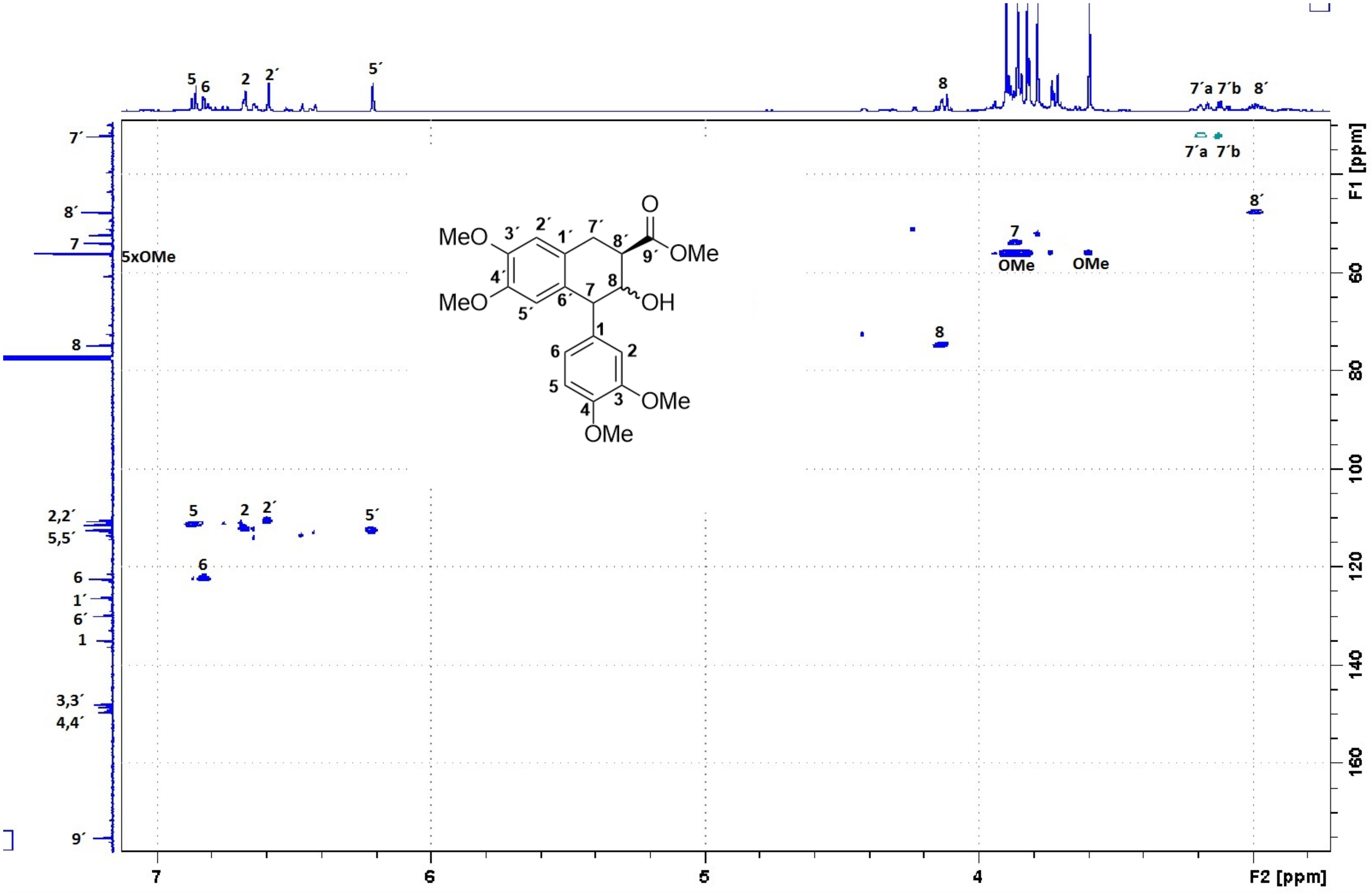

Supplement: Supplementary file 1 [file molecules-25-01160-s001.pdf]
